# Current biomarkers and treatment strategies in Alzheimer disease: An overview and future perspectives

**DOI:** 10.1016/j.ibneur.2023.11.003

**Published:** 2023-11-30

**Authors:** Ritesh P. Bhole, Rupesh V. Chikhale, Karishma M. Rathi

**Affiliations:** a Department of Pharmaceutical Chemistry, Dr. D. Y. Patil institute of Pharmaceutical Sciences & Research, Pimpri, Pune, India; b Dr. D. Y. Patil Dental College and Hospital, Dr. D. Y. Patil Vidyapeeth, Pimpri, Pune 411018, India; c UCL School of Pharmacy, London, United Kingdom; d Department of Pharmacy Practice, Dr. D. Y. Patil institute of Pharmaceutical Sciences & Research, Pimpri, Pune, India

**Keywords:** Alzheimer's disease, Biomarkers, Treatment, Drug targets, Drug therapy, Future scope

## Abstract

Alzheimer's disease (AD), a progressive degenerative disorder first identified by Alois Alzheimer in 1907, poses a significant public health challenge. Despite its prevalence and impact, there is currently no definitive ante mortem diagnosis for AD pathogenesis. By 2050, the United States may face a staggering 13.8 million AD patients. This review provides a concise summary of current AD biomarkers, available treatments, and potential future therapeutic approaches. The review begins by outlining existing drug targets and mechanisms in AD, along with a discussion of current treatment options. We explore various approaches targeting Amyloid β (Aβ), Tau Protein aggregation, Tau Kinases, Glycogen Synthase kinase-3β, CDK-5 inhibitors, Heat Shock Proteins (HSP), oxidative stress, inflammation, metals, Apolipoprotein E (ApoE) modulators, and Notch signaling. Additionally, we examine the historical use of Estradiol (E2) as an AD therapy, as well as the outcomes of Randomized Controlled Trials (RCTs) that evaluated antioxidants (e.g., vitamin E) and omega-3 polyunsaturated fatty acids as alternative treatment options. Notably, positive effects of docosahexaenoic acid nutriment in older adults with cognitive impairment or AD are highlighted. Furthermore, this review offers insights into ongoing clinical trials and potential therapies, shedding light on the dynamic research landscape in AD treatment.

## Highlights

• Alzheimer's disease lacks a definitive ante mortem diagnosis.
• This review article addresses the issue by summarizing AD biomarkers and treatments.
• It covers current treatment options and future therapeutic approaches.
• Discusses promising targets and strategies like Aβ modulation, Tau aggregation, HSP, oxidative stress, inflammation, metals, and ApoE modulation.
• Encourages further research in AD treatment.

## 1 Introduction

Alzheimer's disease is a most familiar cause of dementia in elder individuals, one of the central nervous system's progressive degenerative disorders. Alois Alzheimer first identified AD as a distinct type of dementia in 1907 (Newman et al., 2007). Emile Kraepelin described AD as a "distinct dementing illness" in 1910, while Schorer offered proof that dementia could have a "organic" aetiology in 1985 (Schorer, 2019; Small and Gandy, 2006). In the advanced stages of Alzheimer's disease, reactive astrocytes are observed to be closely linked or in close proximity to the amyloid plaques and tau pathological accumulations in postmortem brain tissues (Amit et al., 2021). However, major dementia in later years was either dismissed as an inevitable final stage of healthy ageing or else attributed to "cerebral atherosclerosis" for decades after that (Katzman, 1986; Small and Gandy, 2006). Pathologically,theaccumulation of amyloid beta peptides and the development of intraneuronal connections due to hyperphosphorylation of axonal Tau proteins are at least two different processes that cause dementia in AD patients (Ray and Lahiri, 2009). No definitive ante mortem diagnosis is currently available confirmation for pathogenesis of AD. To establish diagnosis the current procedures of choice are registry for AD (CERADneutric plague density) scoring and Braakneurofibrillarystaging. They provide estimate of disease severity and complexity (Beach et al., 2012; DeTure and Dickson, 2019); (Fillenbaum et al., 2008; Walton and Dodd, 2007). On January 4, 2011, President of the USAObama signed the National Alzheimer's Project Act into law recognising the seriousness of AD and demonstrating the country's commitment to making AD a national priority (Alzheimer’s association).acknowledging the urgent need for increased research, improved healthcare strategies, and heightened public awareness to address this pressing public health issue (Alzheimer’s Association). Understanding both the historical evolution and the current diagnostic and pathological landscape of AD is paramount for shaping effective interventions and fostering progress in alleviating the burdens imposed by this devastating disease.

## 2 Epidemiology

**Fig. 1:**
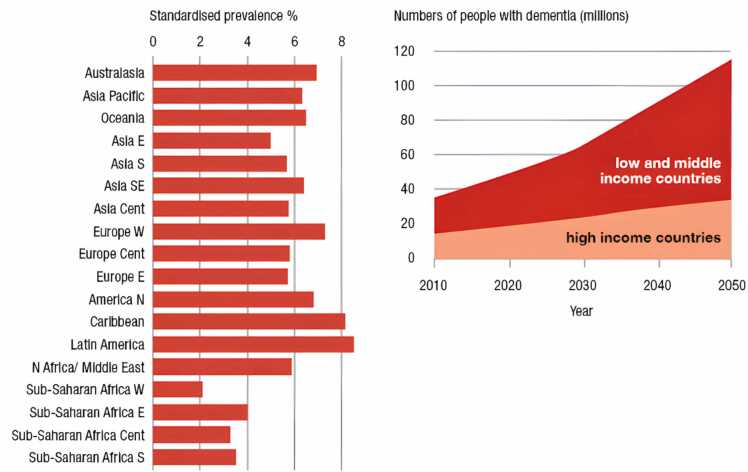
Over the age of 60, according to the GBD region (%) the estimated prevalence of dementia among people, is standardised for the population of Western Europe. (Hilal S).

In developed countries after cancer, CVDs, and stroke, AD is the 5^th^ importantreason of death (Ray and Lahiri, 2009). The 2020ADstatistics showthatin 2050therecouldbe 13.8 million AD patients inthe United States,asignificant increasefrom the current 5.8million (Zhang et al., 2021). In the future, our healthcare and social and economic systems will not be able to bear the financial burden of AD unless and until significant improvements in the prevention and treatment of AD are made.However, in 2050, the prevalence of disease would decrease by more than 9 million incidents if interventions could prolong the onset or progression of diseases by just one year.(Fig. 1)Therefore, effective strategies to prevent and treat AD are urgently needed (Hampel et al., 2010; Alzheimer’s association update, 2011).

## 3 Etiology

**Fig. 2:**
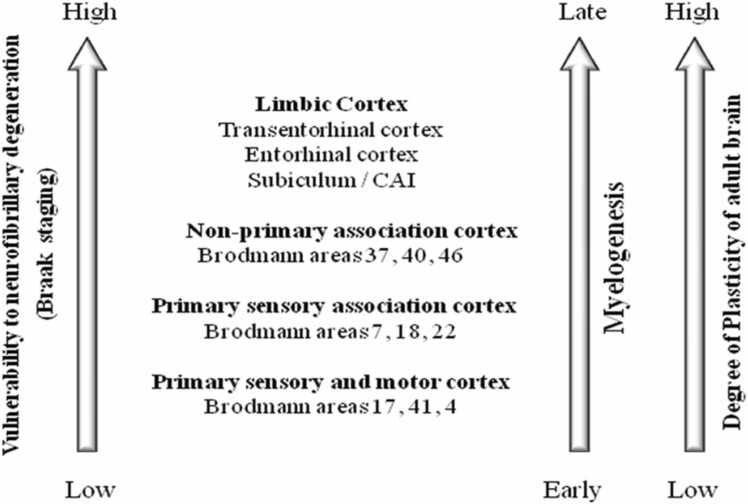
The distribution of vulnerable neurons in AD is consistent when compared to the outline of late ontogenetic development and a high neuroplastic potential in the mature brain, During childhood and adolescence, these brain regions take the longest to develop. The most vulnerable brain structures in both ageing and AD are those very same brain structures.

In affected populations, the disease leads to a progressive decline in cognitive capacity, with episodic memory being the initial target(Artero et al., 2003). However, there is limited understanding of the molecular, cellular, and pathological mechanisms behind the onset of cognitive decline (Lambon Ralph et al., 2003; Newman et al., 2007). Notably, research has shown a strong correlation between (MMSE)Mini-Mental State Examination and (CDR) Clinical Dementia Rating scores and the loss of specific neurons (Bussière et al., 2003; Walton and Dodd, 2007). The transentorhinal region is the first to be impacted, followed by the hippocampus and later the amygdala. Subsequently, neurons in areas such as the motor, sensory motor, anterior cingulate, and occipital cortices face obliteration, resulting in massive cell loss, which is evident in the reduction of gyri and expansion of sulci, marking the affected regions. (Fig. 2).

It is worth highlighting that pyramidal neurons in the neocortex, which project corticocortically, are particularly susceptible to destruction in Alzheimer's disease (AD). This susceptibility has been linked to the observed correlations between MMSE and CDR scores and the loss of these specific neurons (Bussière et al., 2003; Walton and Dodd, 2007). Importantly, neurofibrillary tangle formation and subsequent neurodegeneration are less likely to occur in axons, thalamic projections, primary sensory, and motor (Choi, 1992; Wang et al., 2007).

Based on affecting several areas of the brain the subsequent pathological stages were described as the result of AD analyses with immunocytochemical techniques (Braak et al., 2006):

Stage I: Constricted to the transentorhinal area, minimal involvement.

Stage II: The lesion continuously increases and the entorhinal area is affected by the pathology.

Stage III: The entorhinal region's pathology deteriorates, and lesions spread to the nearby neocortex.

Stage IV: Medial temporal gyrus is affected by neurofibrillary disease.

Stage V: Occipital neocortex is affected by the lesion.

Stage VI: Striate and parastriate regions of the occipital neocortex exhibit lesions (Li et al., 2007, Vasudevaraju et al., 2008).

Genetic changes in AD pathology include mutations in genes (Chai, 2007; Lambert and Amouyel, 2007) including APP Presenilin-1, Presenilin-2 (Bertram and Tanzi, 2012; Van Giau et al., 2019), Apolipoprotein E(Yamagata et al., 2001) and Neuronal sortilin-related receptor (Li et al., 2007; Rogaeva et al., 2007; Vasudevaraju et al., 2008).

## 4 Diagnosis of AD (Woo et al., 2011)

**Table:** 

Laboratory tests	Brain-imaging scan	Recent Advancement
CSF markers: Detection of total-tau, Aβ42 in CSF Additional CSF markers may include phosphorylated tau (p-tau) and the Aβ42/Aβ40 ratio. These markers help in assessing abnormal protein levels associated with AD.	MRI scans:It is useful to identify AD after the occurrence of clinical symptoms or before the occurrence of clinical symptoms.	Diffusion Tensor Imaging Studies And Technology: Allows the mapping of alterations in white matter (WM) microstructure
Urine tests: It is used for measuring the concentration of isoprostane or neural thread protein in urine	PET scans:It uses chemical markers like FDDNP, Pittsburgh compound B.	Quantum Dots: It used astheranostic agent due to high sensitivity, stability and selectivity at a nanoscale range property.
Blood tests:Blood biomarkers for AD encompass not only assessing glucose levels, liver function, and thyroid function but also include novel markers like amyloid-β and tau proteins in blood plasma, neurofilament light chain (NfL),andvarious inflammatory markers.	CT scans: to rule out brain tumors it produces 2D brain images	Artificial Intelligence:A lot of patient data, physiological signals, and sophisticated signal processing are used in computer-aided diagnosis

## 5 Types of AD

It would take more than 50 years for researchers to realise that early-onset (pre-senile or FAD) and late-onset (senile or SAD) dementia are phenotypically identical under the microscope, leading to the unification of the illnesses under the same label (Amaducci et al., 1986). Nevertheless, despite the common name and overlapping characteristics, early and late forms of AD are epidemiologically and etiologically different (Mayeux, 2003; Small and Gandy, 2006).

### 5.1 Sporadic AD (SAD)

Sporadic Alzheimer’s disease occur after 65 years (Ray and Lahiri, 2009). The key genetic risk factor for SAD is the presence of allele ε4 in the apolipoprotein E gene, which is the primary transporter of cholesterol in the brain (Fonseca et al., 2010; (Guerreiro et al., 2012; Kaminsky et al., 2010; Liu et al., 2013; Newman et al., 2007). SAD risk has also been linked to genetic polymorphisms in the transmembrane receptor protein 6 (LRP6), which is associated to low-density lipoprotein receptors (De Ferrari et al., 2007). Aberrant cell cycle, dysregulation of brain energy metabolism brought on by mitochondrial failure, inflammation, oxidative stress, and proteasome dysfunction are a few more complicating elements in the aetiology of AD (van Tijn et al., 2008;(Walsh and Aisen, 2004; Webber et al., 2005). In accordance with the role of glycogen synthesis kinase (GSK)− 3β in AD pathogenesis, Blalock et al. reported that in the hippocampus region, GSK-3βexpression was regulated higher (Ballard et al., 2011, Boonen et al., 2009, Rogaeva et al., 2007).

### 5.2 Familial Alzheimer’s disease (FAD)

Before the age of 65, the APP or PS1 and PS2 genes experience rare autosomal dominant missense mutations, which are the root cause of familial AD or Early Onset AD (Guerreiro et al., 2012); (Ray and Lahiri, 2009); (Yamagata et al., 2001). Aβ40–42 peptides are produced when APP is cleaved deliberately by β-andγ-secretases (Okochi et al., 2013). The Aβ40 peptide is crucial for the maturation of dense cored plaques without directly influencing the course of the disease,although Aβ42 is thought to be the more unstable, (Kumar-Singh et al., 2000).

The β-and γ-secretase cleavage sites are concentrated around the FAD-linked APP mutations, which often cause a less destructive course of the disease than FAD-linked PS mutations. The PS1 and PS2 genes contain several FAD-PS mutations, which are to blame for the majority of FAD cases. Typically, these mutations lead to defective Aβ production, which selectively mediates a rise in the Aβ42/Aβ40 ratio (Bentahir et al., 2006); (Shen and Kelleher, 2007). Even though, the accurate mechanism of Aβ toxicity in either FAD or SAD remains to be elucidated. On chromosome 10 at least one further FAD gene is thought to exist (Ballard et al., 2011). (Fig. 4).

**Fig. 3:**
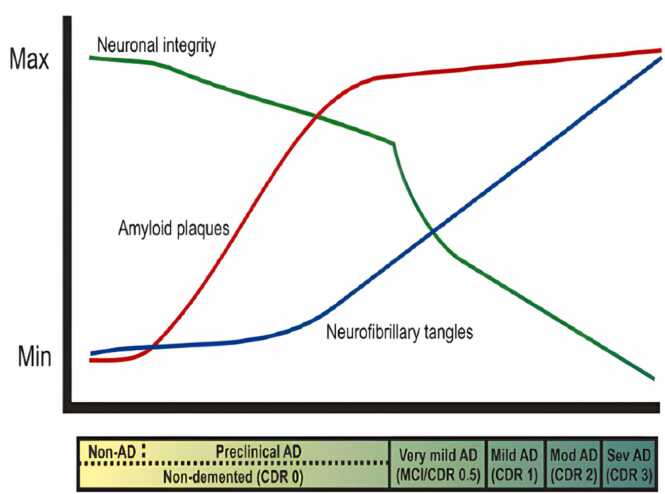
Biomarkers and AD:The relationship between putative biomarker changes and the progression of pathological and clinical phases in AD.The clinical dementia score of 0.5, 1, 2, and 3correspondsto the clinical stages of AD, characterized by progressive dementia described as "very mild/mild cognitive impairment", "mild," "moderate," and "severe." A lot of Aβ (red line), NFTs (blue line), and synapse and in specific brain regions neuronal loss are all associated with these stages (green line) (Tarawneh and Holtzman, 2010).

**Fig. 4:**
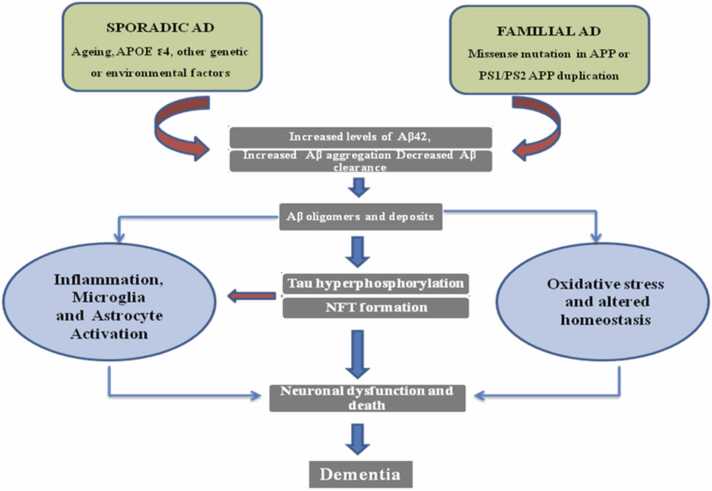
According to the amyloid cascade theory, red arrows represent the primary pathogenic pathway for AD, whereas thinner brown arrows represent the minor contributory pathways. Even though risk factors like age and ApoE genotype both have a significant impact on Aβ aggregation in transgenic models and post-mortem AD brain, there is less evidence to support the theory's applicability to sporadic AD (Philipson et al., 2010).

Familial Alzheimer's disease is characterized by a strong genetic component and is typically associated with mutations in specific genes, including those coding for amyloid precursor protein (APP), presenilin 1 (PSEN1), and presenilin 2 (PSEN2). In contrast, SAD occurs sporadically and is influenced by a combination of genetic and environmental factors. Among these genetic factors, the presence of the ε4 allele in the apolipoprotein E gene has been identified as a significant risk factor, especially in late-onset SAD.

While ε4 is a well-known risk factor for FAD, its association with SAD underscores the complex interplay of genetic and environmental factors in the etiology of Alzheimer's disease. It is important to note that while ε4 increases the risk of developing SAD, it is not the sole determinant, and other factors, such as genetic polymorphisms in genes like LRP6, as well as processes like aberrant cell cycle regulation, mitochondrial dysfunction, inflammation, oxidative stress, and proteasome dysfunction, also contribute to the development of SAD.

In line with the role of glycogen synthesis kinase (GSK)− 3β in AD pathogenesis, research has shown elevated GSK-3β expression in the hippocampus region. Further studies have supported the involvement of additional factors in the complex etiology of SAD.

Familial Alzheimer's Disease (FAD), it is recognized that Aβ42, a specific form of amyloid-beta (Aβ) peptide, is considered to be more unstable compared to Aβ40. This distinction arises from several factors related to the biochemical properties and aggregation behavior of these two peptides.

Peptide Length: Aβ42 is a slightly longer peptide than Aβ40, with two additional amino acids at the C-terminus. These two extra amino acids make Aβ42 more prone to aggregation because they provide additional opportunities for intermolecular interactions that can lead to the formation of larger and more insoluble aggregates.

Aggregation Kinetics: Aβ42 has been observed to exhibit faster aggregation kinetics compared to Aβ40. This means that Aβ42 molecules tend to come together and form aggregates more rapidly. The rapid aggregation of Aβ42 is thought to contribute to the formation of neurotoxic plaques in the brains of individuals with FAD.

Structural Differences: Aβ42 has a propensity to adopt certain structural conformations, such as beta-sheet structures, that are associated with amyloid fibril formation. These structural characteristics can lead to the assembly of insoluble aggregates, which are a hallmark of Alzheimer's disease pathology.

Neurotoxicity: Research has suggested that Aβ42 aggregates may be more neurotoxic than Aβ40 aggregates. The specific molecular mechanisms underlying this increased neurotoxicity are still an area of active investigation, but it is believed to be related to the size and stability of Aβ42 aggregates.

It's important to note that the precise mechanisms by which Aβ peptides contribute to the pathogenesis of Alzheimer's disease, whether in FAD or SAD, are complex and continue to be the subject of intensive research. The greater instability and propensity for aggregation of Aβ42 compared to Aβ40 are among the factors that make it a focus of investigation in understanding the disease process in FAD.

## 6 Drug targets in AD

### 6.1 Biomarkers for AD

An ideal AD biomarker would identify key neuropathological characteristics, have diagnostic sensitivity for AD over 80%, and specificity for differentiating AD from other dementias above 80%. The use of biomarkers may make it easier to accurately identify AD at its very early, even pre-clinical illness stage. Researchers have discovered chemicals that may be connected to cell death in AD in the cerebrospinal fluid, plasma, and urine thus far (Ankarcrona and Winblad, 2005).

The progression of AD can be tracked using a variety of biomarkers. (Fig. 3) As in the case of death or recurrence in cancer, the use of mortality endpoints is not practical. There are no more unambiguous events that could be assessed due to the extremely high sample sizes or follow-up periods that would be necessary (Thomas et al., 2000). As a result, clinical assessments of cognitive or functional status as well as overall evaluations are currently used as the major endpoints in AD trials (Coley et al., 2009, Ray and Lahiri, 2009, Vincent and Maiese, 2000).

**Aβ Peptides (Aβ (1−40) and Aβ (1−42)): Aβ peptides are among the most widely studied and important biomarkers for AD. Here's why they are significant:**

**Aβ (1−40) and Aβ (1−42) Levels**: Elevated levels of Aβ (1−42) and altered Aβ (1−42)/(1−40) ratios in cerebrospinal fluid (CSF) and plasma are considered indicative of AD. Aβ (1−42) is particularly relevant because it has a greater tendency to aggregate and form plaques compared to Aβ (1−40).

**Amyloid imaging**: Aβ imaging using positron emission tomography (PET) can visualize amyloid plaques in the brain. This imaging technique is valuable for diagnosing AD and tracking the progression of amyloid deposition.

**Research and drug development**: Aβ peptides are essential in AD research and drug development. They are targeted in clinical trials, and therapies aim to reduce Aβ production or clear Aβ aggregates from the brain.

**Early detection**: Changes in Aβ levels often precede clinical symptoms of AD. Therefore, Aβ biomarkers are crucial for early detection and intervention when potential treatments might be more effective (Kitazawa M et al., 2012).

**Amyloid precursor protein (APP) and Aβ peptides**.

APP is a mammalian type I transmembrane protein. The endoplasmic reticulum (ER) first produces APP, which is then successively digested by β - or γ-secretases to produce Aβ. As a result of these cleavages, secreted APP and the APP intracellular domain,which contains 99 amino acid residues (C99), are produced in soluble extracellular fragments (Perry et al., 1987). As secretase proceeds to cleave C99, various Aβ peptides with various numbers of amino acid residues are produced (Vasudevaraju et al., 2008). The most prevalent type of Aβ, Aβ (1−40), has forty amino acid residues and is less harmful for the onset of AD. Though, Aβ with 42 residues of amino acids Aβ (1−42) is fibrilar in nature and forms aggregates that are pathognomonic for AD, despite being synthesised in much lower levels (Ray and Lahiri, 2009).

It's important to note that while Amyloid Precursor Protein (APP) itself is not a biomarker, the cleaved fragments, especially Aβ (1−40) and Aβ (1−42), are widely used as biomarkers to detect and characterize AD.Additionally, the Aβ1–40/Aβ1–42 ratio serves as another valuable biomarker in AD, offering insights into the amyloid burden in the brain and aiding in the diagnostic process.

**Fig. 5:**
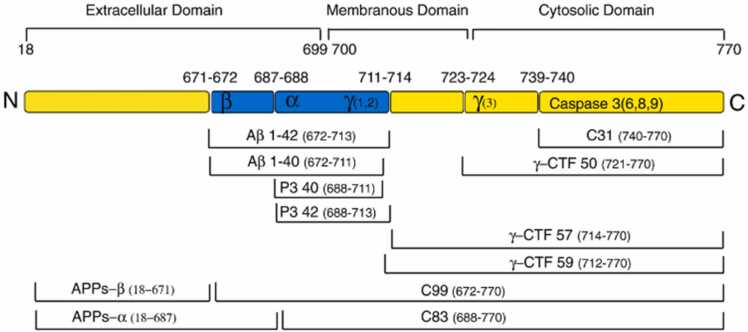
The interior fragments of APP770 and the peptide connections of α-, β-, and γ-secretases split are shown in schematics. The C-terminal fragments of the APP linked to the membrane C83 and C99 were generated when the α-secretases separated the APP. CTF represents the C-terminal fragment; γ-CTF represents the C-terminal fragment broken down by γ-secretase. APPs-α and APPs-β are cut by α- and β-secretases and are soluble N-terminal segments of APPs (Kaminsky et al., 2010).

This multi-faceted approach to biomarker research, including the Aβ1–40/Aβ1–42 ratio, offers promising avenues for the early detection and characterization of Alzheimer's disease as shown in (Fig. 5).

**Fig. 6:**
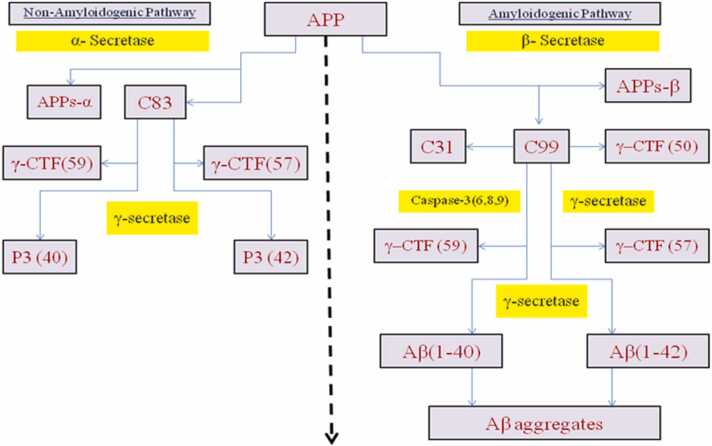
Schematic pathways of APP proteolytic processing.

"APP is associated with mitochondrial dysfunction in neurons affected by AD."(Anandatheerthavarada and Devi, 2007). In contrast to the control brain, they found that the full-length APP and the C-terminal APP species (which lack the Aβ domain) accumulated in the mitochondria of the AD-associated brain (Reddy, 2009). (Fig. 6).

### 6.2 Amyloid β (Aβ)

**Fig. 7:**
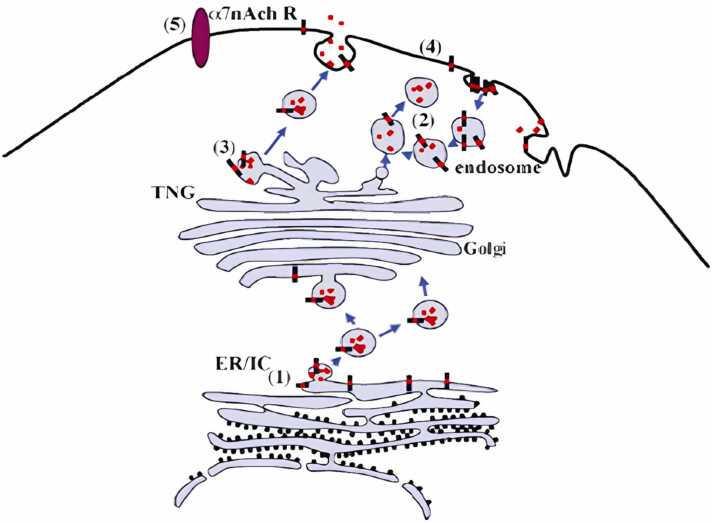
Aβ's origin and source. (1) In the ER lumen, β- and γ-secretases can cleave APP to produce Aβ42. (2) Aβ can damage the lysosomal membrane and leak into the cytoplasm. when it is present in the endosome/lysosome system, (3) Passive Aβ leaking through any part of the secretory route. either (5) Inside the cell, Aβ binds to surface receptors such as 7AchR (4) Into the cytosol Aβ passively diffuses the plasma membrane (Vasudevaraju et al., 2008).

**Fig. 8:**
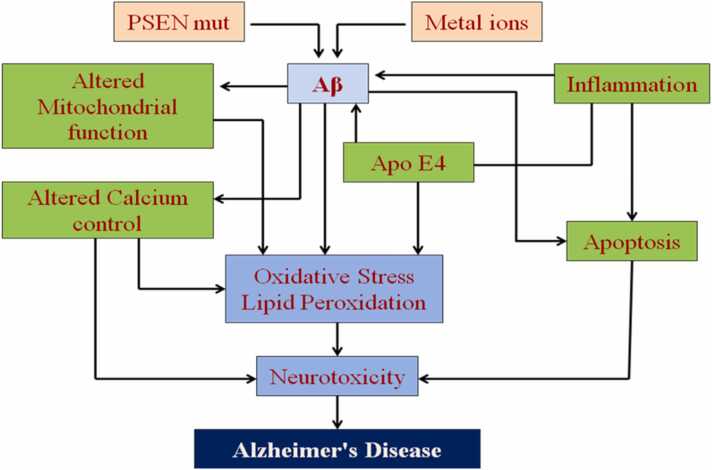
Aßamyloidscan be formed by increased synthesis (e.g., psenilline mutations) or reduced elimination.(e.g.,APOE4).Aß' oligomerization leads to the production of oxidative stress itself through induction of several other interaction mechanisms.

25 years ago,Aβ was first identified and sequenced from the meningitis blood vessels of AD and Down syndrome patients (Fig. 7, Fig. 8) (Drummond et al., 2022); (Glenner and Wong, 1984); (Masters et al., 2006). Aβ proteolytic residues of APP processing have no clear physicochemical characteristics known in advance (Murphy and LeVine, 2010);H. (Zhang et al., 2012) and integrated surface-enhanced laser decomposition and ionization time-of-flightmass spectrometry and monoclonal antibodies such as 6E10 have been used to identify peptides Aβ2–14, Aβ1–17, Aβ1–18, Aβ1–33, Aβ1–34, Aβ1–37, Aβ1–38, Aβ1–39, Aβ1–40, and Aβ1–42, in cerebrospinal fluid of AD patients (Maddalena et al., 2004).

The temporal lobe of young drug abusers, AD brains, Down’s syndrome patients accumulation of Aβ is detected.(D’Andrea et al., 2001);(Gouras et al., 2000);Kitazawa et al., 2012),and (Vasudevaraju et al., 2008) it is the primary amyloid component of extracellular senile plaques, found in AD patient's brain that cause gradual neuronal degeneration(H. (Zhang et al., 2012). Recently, In the mitochondrial membranes of the neurons in postmortem brain tissues from AD patients, Aβ monomers and oligomers were discovered (Crouch et al., 2006);(Devi et al., 2006). According to Hansson Petersen et al., the outer membrane machinery's translocase is how Aβ is delivered into mitochondria (Hansson Petersen et al., 2008, Reddy, 2009). CSF Aβ is transferred from the arachnoid granulations to the venous circulation. Several enzymes can break down Aβ derived from APP (Cummings, 2011).

Transition metals are such efficient precipitators that the binding of a single copper or zinc atom is believed to produce Aβ precipitation (Atwood et al., 2000). In contrast, Aβ is monomer, β-helix-shaped, and cannot aggregate if no metal exists. In recent years, it has been shown that Aβ is a very efficient chelator for transitional metal ions (such as copper) (Atwood et al., 2000)whichare more efficient oxidation catalysts. Aβ1–42 is more sensitiveto metal ions than Aβ1–40 (Kontush, 2001).

It's important to note that while Amyloid Precursor Protein (APP) itself is not a biomarker, the cleaved fragments, especially Aβ (1−40) and Aβ (1−42), are widely used as biomarkers to detect and characterize AD.Additionally, the Aβ1–40/Aβ1–42 ratio serves as another valuable biomarker in AD, offering insights into the amyloid burden in the brain and aiding in the diagnostic process (Atwood et al., 2000a).

This multi-faceted approach to biomarker research, including the Aβ1–40/Aβ1–42 ratio, offers promising avenues for the early detection and characterization of Alzheimer's disease.

There are few hypothetical routes that can lead to the accumulation of Aβ (Lin et al., 2001):

If insoluble Aβis generated in the endoplasmic reticulum, it might be identified as a misfolded protein and returned to the cytoplasm by reverse translocation. Ubiquitinating and delivering the misfolded proteins to the proteasomes for destruction. Ineffective clearance and destruction of Aβ could cause Aβ to accumulate since the proteasome activity declines with ageing.
Internalised Aβ is taken up by endosomes. It has been hypothesised that Aβ can make lysosomes' membranes more permeable. The Aβ in the lysosome system could harm the membrane and seep into the cytosol.
Passive leaking along a secretory route component of any kind (Cole et al., 2005); (Nagele et al., 2002).

The statements regarding Aβ40 may seem contradictory at first glance but can be reconciled by considering the distinct roles of different Aβ peptide isoforms in Alzheimer's disease (AD) pathogenesis.

Aβ40 and Dense Cored Plaques: It is indeed true that the Aβ40 peptide plays a crucial role in the maturation of dense cored plaques, which are a hallmark pathological feature of AD. These plaques primarily consist of aggregated Aβ peptides, including Aβ40. The presence of Aβ40 is instrumental in the formation and maturation of these plaques in the brain.

Role in Disease Onset: While Aβ40 is involved in the formation of plaques, it is generally considered to be less neurotoxic than another variant, Aβ42. Aβ42 is thought to be more prone to aggregation and is often associated with greater toxicity to neurons. This is why Aβ42 is often viewed as a key contributor to the early stages of AD onset.

So, the distinction lies in the role that Aβ40 plays in the maturation of dense cored plaques, which are a pathological feature observed in the brains of individuals with AD. However, the specific neurotoxicity and impact on disease onset are more strongly associated with Aβ42, a different variant of the Aβ peptide. Both Aβ40 and Aβ42 are important components in understanding AD pathogenesis, but their contributions and effects differ.

In summary, while Aβ40 is crucial for plaque formation, it is considered less harmful in terms of disease onset compared to Aβ42, which is often linked to the early stages of Alzheimer's disease (Monji et al., 2001).

**Amyloid β (Aβ) degradation enzymes:**

Several enzymes are involved in the degradation of Aβ peptides derived from amyloid precursor protein (APP). These enzymes play a critical role in regulating Aβ levels in the brain. While numerous enzymes are implicated in Aβ metabolism, here are a few notable examples:

**Neprilysin**: Neprilysin, also known as neutral endopeptidase (NEP), is an enzyme that degrades Aβ peptides. It cleaves Aβ at specific sites, reducing the accumulation of toxic Aβ species in the brain. Neprilysin has been the focus of research for its potential role in AD therapeutics [66].

**Insulin-degrading enzyme (IDE)**: IDE is another enzyme responsible for Aβ degradation. It has a broad substrate specificity and can cleave Aβ peptides among other substrates. IDE dysfunction has been associated with impaired Aβ clearance in AD [66].

**Matrix metalloproteinases (MMPs):** Certain members of the matrix metalloproteinase family, such as MMP-2 and MMP-9, have been implicated in Aβ degradation. They can cleave Aβ peptides and contribute to their clearance [66].

Endopeptidase-24.15 (ThimetOligopeptidase): This enzyme has been shown to degrade Aβ peptides, particularly Aβ (1−42) [67].

These enzymes, among others, are part of the complex regulatory network responsible for maintaining Aβ homeostasis in the brain. Dysregulation of these enzymes can lead to Aβ accumulation, which is associated with Alzheimer's disease pathology.

#### 6.2.1 Mechanism of Aβ toxicity

The production of reactive oxygen species, such as hydrogen peroxide, nitrogen oxides, superoxides, highly reactive hydrogen atoms (Fonfria et al., 2005);(Kardos et al., 2018);(Lamoke et al., 2015); (Malyshev et al., 2005), intracellular calcium accumulation excitotoxicity (De Simone et al., 2019, Malyshev et al., 2005, Yue et al., 2022) resorption of membrane fluids, aging of the cytoskeleton,inflammation, and metal homeostasis are several mechanisms that have been proposed to explain the toxic effects of fibrous Aβ neurotoxicity (Barnham et al., 2004); (Bush, 2003);(Hardy and Selkoe, 2002). These events combine to form similar pathways of synaptic disturbance, and necrosisleading to progressive loss of certain neuronal cell populations (Gaeta and Hider, 2005, Guerreiro et al., 2012, Kaminsky et al., 2010, Kontush, 2001, Masters et al., 2006).

There is strong evidence that Aβ triggers apoptosis when severe mitochondrial damage occurs. Cytosol cysteine protease can be induced when Aβ interrupts mitochondrial caspase-9 and-3 (Alnemri et al., 1996). Pro-Caspase-9 activates Caspase-9after binding to Apaf-1, which requires cytochrome c and ATP in the cell. Caspase-9 can then separate and activate caspase-3. Caspase-3can directly participate in DNA fragmentation (Jänicke et al., 1998) such as cytoplasmic cleavage of DNaseactivated by Caspase, resulting in apoptosis (Kaminsky et al., 2010).

#### 6.2.2 Aβas a redox-active

Aβ has two important sites for its oxidation activity. (Fig. 10) The 1stsite is located in the N-terminal hydrophilic part of the peptide, composed of 3 residues of histidine (6, 13and 14) and tyrosine (at position 10), all of which are effective in chelating transition metal ions (Løvstad, 1987). Theoretically, metal-catalyzed biomolecule oxidation might be prevented by the chelation of transition metals in a redox-inactive state at this location. One methionine residue at position 35 makes up the 2nd site, which is located in the lipophilic C-terminal region of protein Aβ (Drazic et al., 2013, Hiller and Asmus, 1981).

**Fig. 9:**
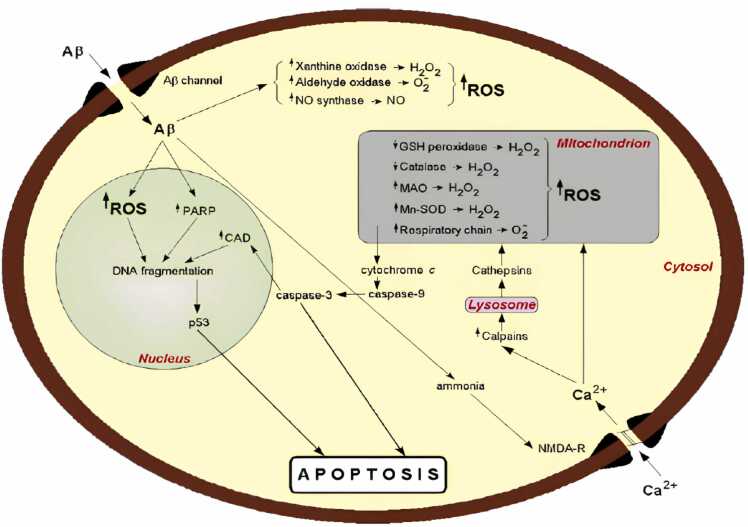
SchemeticIllustration:The subsequent sequence of intraneuronal activity following Aβ effect(Kaminsky et al., 2010).

**Fig. 10:**
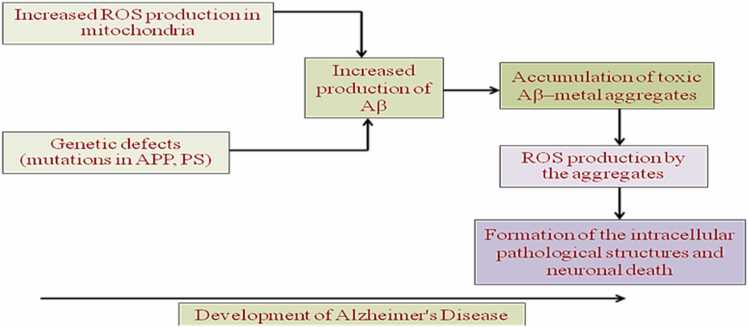
Development of Alzheimer Disease.

It is significant to note that, contrary to a recent proposal, Met35 oxidation by metals is a more frequent process than its extemporaneous metal-independent radicalization through the production of the sulphur-centered radical of Aβ. In addition to catalysing additional oxidation of biomolecules, reduced metal ions can also create extremely reactive hydroxyl radicals from H_2_O_2_ (Cadenas and Davies, 2000). Another good pattern is the oxidation of hydrogen amino impurities observed when Aβ was incubated with some spin traps in a laboratory buffer and traces of transition metals were found(A et al., 2001). Met35 residues of Aβ can reduce metal oxidation by providing redox cycles necessary for hydroxylamine oxidation (Kontush, 2001).

#### 6.2.3 Aβas a pro-oxidantagent (Brown et al., 1997)

His 13 seems to be essential for the aggregation of Aβ (Istrate et al., 2016),andMet35 requires the antioxidative activity of Aβ. Considering the distinctive redox properties of Aβ, it is possible to understand the three elements necessary for fibrillation, transition metals, and Met35 presence for Aβ's prooxidative activity. In order to generate ROS as an oxidative agent,Aβ must first bind to metal binding sites and then reduce them to reduction sites. Fibrillation is likely to achieve this function by forming a complex where metal atoms bound to the N-terminal part of an Aβ molecule can simultaneously be obtained for reduction residues of Met35. The metals must be placed near the reduced agent to reduce. Transitional metal ions that have been reduced can participate in additional redox processes to produce various types of free radicals (Mayes et al., 2014).

### 6.3 α-secretase

The separation of theAPP outside the central nervous system is preferred by α-secretase.The proteinase activity of α-secretase (Ling et al., 2003) is mediated by membrane-bound proteins, members of the ADAM (decomposition and metalopolyprase) family. It also separates APPs from the Aβ sequence itself between 16 and 17 amino acids, generating the soluble ectodomain of APPs and the membrane-bound carbon dioxide terminal fragment, APP-CTFα. The latter fragment is further processed byα-secretase or destroyed by lysosomes and produces short hydrophobic peptide groups, including Aβ-17–40 and Aβ-17–42. The production of APPs by α-secretase is believed to be protective in the context of AD because the enzyme is broken up in the sequence of Aβ and thus prevents the production of it. However, the importance of this postulate depends on the many unexplored facets of the biology of APPs and Aβ.

In regulating α-secretase activity, another indirect way of encouraging APP separation via α-secretase is to stimulate one or more signal transmission pathways. Since protein kinase C, tyrosine kinases, and calcium-mediated pathways are all known to play a role in controlling α-secretase activity. Retinoic acid derivatives may be used to indirectly stimulate the cleavage of APP by α-secretase by increasing the transcription of ADAM10 (a disintegrin and metalloprotease 10) (Smith et al., 1998).

### 6.4 β–secretase

In 1999, the molecular cloning of β-secretase was independently reported by five different research teams, and as a result, this enzyme has gone by a number of names, including β-site APP cleaving enzyme, aspartyl protease 2, and memapsin2 (Ling et al., 2003). In the rough endoplasmic reticulum, β–Secretase is synthesized and transported to the Golgi network, such as the pre-BACE-1, with a molecular mass of about 65 kDa. Itcatalyzes the initial step of C99 separation to produce Aβ peptides. To all forms of Aβ, including the pathogenic 42 amino acid version of Aβ, it can be inhibited β-secretase (Aβ42), which may be a prime treatment target for AD. The therapeutic target forβ-Secretase in AD has a number of benefits (Ling et al., 2003).

First, As demonstrated by the lack of Aβ in β-secretase gene deletion it commences the production of Aβwithout a compensatory activity (Cai et al., 2001); (Luo et al., 2001); (Roberds et al., 2001). All of the detrimental downstream processes in the development of AD can be stopped by inhibiting β-secretase. Second, aβ-secretase inhibitor medication intended at partial reduction of the protease activity produced very mild behavioural changes in mice when the β-secretase gene was deleted, suggesting the potential of patient safety.The lack of a severe phenotype also shows that the clinical use of β-secretase inhibitors does not raise any significant safety concerns regarding the removal of processing for other β-secretase substrates. Adult mice's brains were directly infused with β-secretase inhibitor, which significantly decreased Aβ levels but had no effect on the myelination of the axons (Sankaranarayanan et al., 2008). Finally, there is a significant chance of producing a clinically relevant β-secretase inhibitor in light of past successes in the drug development of HIV protease, which is likewise an aspartic protease (Ghosh et al., 2008).

The discovery of medications for the treatment of AD was made possible by in-depth study of the structure, location, activity, and regulation of β-secretase. For β-secretase understanding the catalytic activity of the enzyme and creating inhibitors based on its crystal structure was essential(L. (Hong et al., 2000). In the mammalian brain, acidic organelles of the endosomes and trans-Golgi network, β-secretase mRNA has the highest level of expression (Vassar et al., 1999). The make-up of the lipid raft domains in the membrane bilayer controls β-secretase activity and access to substrates (Bolognesi et al., 2009).

### 6.5 γ-secretase

Genetic, pharmacological protein, and cell biology investigations have all come together to show that γ-secretase is a multi-subunit aspartyl protease that cleaves APP. Two aspartate residues, which are necessary to catalyse the hydrolysis of a peptide bond, are a characteristic feature of aspartyl proteases (Haass and Steiner, 2002). The catalytic centre of γ-secretase is composed of presenilins PS1 and PS2, which are two presenilins. The γ-secretase complex also needs the three auxiliary proteins anterior pharynx-defective 1, nicastrin, to be complete De Strooper ;Takasugi et al., 2003;Wolfe and Kopan, 2004;Woo et al., 2011;Yu et al., 2000 (De Strooper, 2003, Takasugi et al., 2003, Wolfe and Kopan, 2004, Woo et al., 2011, Yu et al., 2000). They appeared to play a role in the complex's evolution and stability.

The particular intramembrane proteolysis activity of γ-secretase sets it apart from other secretases like α - and β-secretase; they can pull off the remarkable feat of employing water to cleave the protein present in water hating environment of the cellular membrane (Wolfe and Selkoe, 2002).

**Fig. 11:**
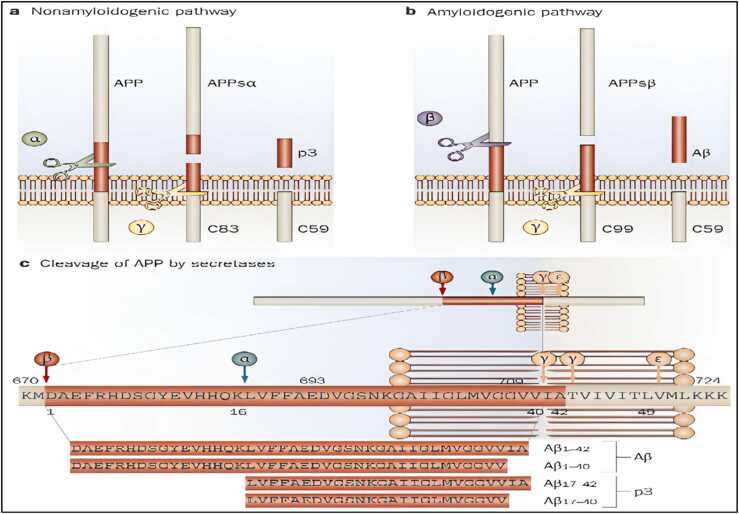
Cleavage of APP by secretases. In the nonamyloidogenic pathway, the APP's ectodomain is first released when α-secretase cleaves the protein within the Aβ sequence. When β-secretase cleaves APP at the amino terminus of the Aβ peptide and releases the APPs ectodomain, the amyloidogenic pathway is started.By digesting APP at two separate sites by γ-secretase, Aβ and p3 fragments of various lengths are created. Abbreviations: C83, carboxy-terminal fragment 83, 59, 99; Aβ, APP, APPsα, APPsβ (De Strooper et al., 2010).

The fact that γ-secretase conversely cleaves the substrate's membrane "stub" after ectodomain "shedding" is particularly intriguing from a therapeutic standpoint. A cleavage known as the " ε-cleavage" appears to cleave the substrate within the transmembrane domain close to the cytoplasmic border of the membrane. The intracellular domain is liberated from the membrane by the ε-cleavage, allowing it to perform an intracellular signalling function. Notch translocates to the nucleus after γ-secretase processing, where it binds to numerous intracellular proteins and controls transcription. The amino-terminal region of the substrate's membrane stub is secreted after the substrate is first cut at the ε-cleavage site and then again within the transmembrane domain. This successive cleavage of APP as shown in (Fig. 11) by γ-secretase results in Aβ and appears to be a crucial factor in deciding whether or not a person will develop AD (De Strooper et al., 2010; Huang et al., 2006).

### 6.6 Tau protein

The integral part of NFTs, a hallmark of AD and other illnesses such as frontotemporal dementia associated to chromosome 17, is the microtubule binding protein tau (Schenk et al., 2006). Tau can no longer stabilise microtubules because it is broken down into paired-helical fragments (PHF) and protein aggregates (NFTS) when it is hyperphosphorylated. Neurodegeneration may occur if these latter, which are crucial for intracellular trafficking, are harmed. Tauopathies are diseases that are characterised by the presence of NFTs (Bulat and Widmann, 2009).

Tau is a nuclear protein that is found in both neuronal and non-neuronal cells (Thurston et al., 1997) and associates with the mitotic cell's nucleolar organiser region (NOR). Ribosomal DNA close to the centromeres makes up the NOR in the chromosome. Both single-stranded DNA and double-stranded DNA can attach to tau, as has been demonstrated (Hua and He, 2003, Krylova et al., 2005). Tau that has been phosphorylated bonds to DNA more firmly than tau that has not been phosphorylated because the former's melting point is greater. Tau is phosphorylated in both the cytoplasm and the nucleus in similar amounts. Tau is phosphorylated in the cytoplasm before being taken into the nucleus (Bulat and Widmann, 2009, Greenwood and Johnson, 1995, Rogaeva et al., 2007).

Tau protein has been shown to be a strong, Ab-independent activator of the traditional complement system. Tau can therefore boost the production of cytokines and inflammatory reactions. The potential of Aβ deposits and extracellular NFTs to activate complement provides a mechanism for starting and maintaining low-level chronic inflammatory responses that can accumulate during disease, as these injuries occur in early-preclinical to late AD stages(Rojo et al., 2008);(Shen et al., 2001). Tau appears to be largely intact in AD and other tauopathies (Goedert, 1996, Grundke-Iqbal et al., 1986);Grundke-Iqbal, Iqbal, Tung, et al., 1986; (Tetsuya et al., 2022). But immunohistochemical analysis has revealed that tau in AD NFT is shortened at both Glu-391 and Ser-421(Gamblin et al., 2003); (Oakley et al., 2020). In cultured cells, it has been demonstrated that these shortened taus are connected to apoptosis (Fig. 9) (Fasulo et al., 2000); (Rissman et al., 2004).

(GSK)− 3, cyclin-dependent protein kinase (CDPK)− 5, casein kinase-1, protein kinase A,C, calcium and calmodulin-dependent protein kinase-II, are the major kinases, whose activity and protein levels are said to be increased in AD and other tauopathies (Biernat et al., 1993, Ferrer et al., 2005, Harris et al., 2004, Ishiguro et al., 1988, Lordén et al., 2022, Oba et al., 2020, Savage et al., 2002).

Metal ions including zinc, copper, and iron can activate several of the kinases that phosphorylate tau, leading to hyperphosphorylation and the eventual dissociation from the microtubules (An et al., 2005); Björkdahl et al., 2005). The AD brain's tangle-bearing neurons have been additionally found to be filled with zinc(Björkdahl et al., 2005; (Cruz and Tsai, 2004); (Ostrakhovitch et al., 2002); (Scaltriti and Baselga, 2006)(J. H. (Suh et al., 2005). Several tau-kinases activated by zinc also phosphorylate NFs at various locations(Björkdahl et al., 2005; (Cruz and Tsai, 2004);(Ostrakhovitch et al., 2002);(Scaltriti and Baselga, 2006).

### 6.7 Tau kinases

Proline-directed protein kinases that target serine or threonine-proline motifs are the main tau kinases. GSK-3β and cdk5 are well-known enzymes connected to neurofibrillary disease in AD (Liu et al., 2005); (Takashima, 2006). CaMKII, MARK, PKA, PKC, SGK, and p70S6are the examples of non-proline-directed kinases are that phosphorylate extra tau protein sites.Tau protein hyperphosphorylation can also result from the suppression of protein phosphatases (Liu et al., 2005); (Wang et al., 2007). In general, tau protein site-specific phosphorylation appears to control different functions in neurofibrillary pathogenesis, including (i) impairing tau binding to microtubules, (ii) By proteases making tau protein vulnerable to truncation, and (iii) Aggregation to tangles, promoting self-assembly into filaments (Dickey et al., 2008); (Gendron and Petrucelli, 2009); (Iqbal et al., 2009); (Pei et al., 2008);(Sankaranarayanan et al., 2008);(Schenk et al., 2006);(Wang and Liu, 2008);(Wu et al., 2010).

#### 6.7.1 Glycogen synthase kinase-3β

In the Wnt signaling pathway, GSK-3β exists as two homologs, GSK-3α and GSK-3β, which are now thought to function identically (Boonen et al., 2009, Doble et al., 2007). In the brains of Alzheimer's patients GSK-3β expression is seen in the cytoplasm of neurons, and it occurs at the same time as the onset of neurofibrillary alterations.

The phosphorylation of tau by this kinase is supported by a large body of research. Kinesin and collapsing mediating protein 2 may both be phosphorylated, which may result in reduced axonal outgrowth and impaired intracellular transport, respectively. Aβ exposure can activate GSK-3β at the cellular level in cultured hippocampus neurons (Goutte et al., 2002). Lithium and sodium valproate, are directed against GSK-3β and the GSK-3β inhibitor, can reduce cellular damage brought on by Aβ (Chong et al., 2005, Davies and Koppel, 2009, Mondragón-Rodríguez et al., 2012, Themoteo et al., 2023).

GSK-3β has been demonstrated to control APP processing (Phiel et al., 2003); (Ryder et al., 2003) and, however, to allow Aβ peptides to activate it (Sayas and Ávila, 2021)or the APP intracellular domain (Balaraman et al., 2006), also to implicate in neuronal apoptosis (Huang et al., 2006, Jope and Bijur, 2002). The phosphorylation of numerous substrates by GSK-3 is inhibitory because GSK-3 normally inhibits signalling pathways like the insulin and Wnt pathways. When these pathways are active, GSK-3 is inhibited, allowing the action of downstream effectors. Protein kinase Akt, is activated by insulin and phosphorylates GSK-3 at its N-terminal serine residues preventing it from activating downstream signaling (Chiu et al., 2013, Kitazawa M et al., 2012).

GSK-3β inhibition increases survival in a range of cell types, including neurons, while GSK-3β overexpression causes apoptosis (Bussière et al., 2003;(Huang et al., 2006). GSK-3β inhibitors shield neurons from apoptosis brought on by radiation, C2-ceramide neurodegeneration, and glutamate-induced excitotoxicity(Lie et al., 2005);(Reddy, 2009).

Additionally, GSK-3 can control how APP is processed and how tau is phosphorylated by accelerating APP's cellular maturation(Zheng-Fischhöfer et al., 1998), which is thought to take place during the early stages of AD (Gouras et al., 2000), GSK-3 enhances Aβ release. One of the potential protein kinases that can phosphorylate the tau protein is GSK-3. PHF-containing NFTY is primarily made up of hyperphosphorylated tau. The successive phosphorylation of tau at the locations critical for PHF production appears to need GSK-3 (Lucas et al., 2001). Tau is hyperphosphorylated in hippocampus neurons when GSK-3 is overexpressed in transgenic mice, which causes tau to localise in pre-tangle-like somatodendritic structures (M. (Hong et al., 1997). Lithium, a GSK-3 inhibitor, blocks tau hyperphosphorylation and promotes tau attachment to microtubules, which results in microtubule assembly (Chong et al., 2005, Kim et al., 2003).

GSK-3's ability to hyperphosphorylate tau may necessitate the cleavage of APP by caspase 3. The C-terminal fragment C31 produces by caspase 363 cleaves of APP at the caspase site D720 of the C-terminus (Ostrakhovitch et al., 2002). Once produced, C31 increases tau protein phosphorylation and glycogen synthase kinase-3 expression (Chong et al., 2005, Kang et al., 2002).

**GSK-3 in APP processing**.

PS1, a subunit of secretase, has been shown to interact with GSK-3 by numerous studies (Sun et al., 2002). The role of GSK-3 in the y-secretase complex has not been established, and it is not believed to be a key component of the complex. Nevertheless, numerous studies (Ahn et al., 2005, Phiel et al., 2003, Ryder et al., 2003) have shown that GSK-3 inhibition impairs the processing of APP in both cell culture and animal models (Janicke et al., 1998).

It is crucial to figure out how GSK-3 controls APP processing since doing so could help us better understand how APP processing is controlled generally and help us find new possible therapeutic targets. The lack of direct inhibition of y-secretase by GSK-3 inhibitors raises the possibility that GSK-3 regulates APP access to the secretase complex rather than directly modifying y-secretase activity (Phiel et al., 2003). Compared to direct y-secretase inhibitors, which impair the functionality of numerous y-secretase-dependent proteins, this is a benefit. It has been suggested that GSK-3 phosphorylates APP and PS-1. Although none of these hypothesisedphosphorylations has been shown to take place in living organisms, they both have the potential to control how PS1 and APP interact (Chiu et al., 2013).

#### 6.7.2 CDK5

Multiple sites on tau are phosphorylated by cyclin-dependent kinase 5. Due to the fact that, unlike all other cyclin-dependent kinases, this one is maintained in an active state by the binding of a protein partner known as p25 rather than by phosphorylation, this kinase may be the ideal target from the standpoint of specificity. Three kinds of compounds have been discovered through small molecule screens that block this kinase without requiring the ATP site. One group of these substances included inhibitors that are competitive for tau but non-competitive for ATP. These inhibitors are a prime class of substance for additional research because they may be quite specific (Schenk et al., 2006); (Verghese et al., 2012).

#### 6.7.3 Heat shock proteins and the ubiquitin proteasome system

Heat shock proteins are effective defenders of cells from various types of stress (Androvitsanea et al., 2021); (Pearl and Prodromou, 2006). Based on their attributes, the mammalian hsp group can be divided into numerous families, including the Hsp90, Hsp70, Hsp60, Hsp40, and tiny hsps. Three domains make up the Hsp90 protein: (i) the N-terminal domain, which interacts with client proteins and has a binding site for adenine nucleotides; (ii) the middle segment; and (iii) the C-terminal domain, (Andronesi et al., 2008). The Hsp90 dimer creates a molecular clamp, in which ATP binding promotes conformational changes and the clamp is closed, activating and stabilising the client protein, such as protein kinases.

Mori et al. and Perry et al. (Perry et al., 1987, Riedel et al., 2010) discovered that tau-containing PHFs are ubiquitin-positive shortly after tau was found in NFTs. Hsp90 inhibitors can effectively stop the aggregation of both tau protein and α -synuclein, as shown by therapeutic research (Caccamo et al., 2010, Sittler et al., 2001, Waza et al., 2006). In transgenic AD mice, rapamycin, an autophagy inducer, can reduce Aβ and tau pathology and restore cognitive deficits (Salminen et al., 2011, Schipper, 2000).

### 6.8 Oxidative stress and AD

The fact that AD is an ageing disease is something that many of the theories of AD pathogenesis have neglected to mention. It's significant that this is true even in people who have a genetic risk, such as those who inherit AD via the autosomal dominant gene or with Down syndrome who develop the pathology of AD. Therefore, regardless of genetic predisposition, age is a clear contributor in all cases of AD (Huang et al., 2006); (Zhu et al., 2007).

Risk of oxidative damage in brain (Bulat and Widmann, 2009, Gaeta and Hider, 2005):

1. High O_2_ consumption (20% of basal oxygen intake for the entire body);
2. Critically high ascorbate and iron levels (important for inducing membrane lipid peroxidation);
3. Antioxidant protecting agents are present, but at relatively modest quantities;
4. The propensity for accumulating metals.

It is thought that oxidative stress contributes to both the beginning and development of AD. In SAD, transient hypoxia can result in mitochondrial malfunction, compromised APP cleavage and membrane integrity. During the progression of AD, lipid,protein, DNA oxidation and hydroxylation (Good et al., 1996), protein carbonylation and nitration, AGE products, nitration and free carbonyls have been reported (Aksenov et al., 2001); (Castegna et al., 2003);(Cras et al., 1990);(Williamson et al., 2002) By metal ion reduction, Aβ has been discovered to produce ROS such hydrogen peroxide and cause oxidative damage in neurons. The aggregational condition of the peptides has a significant impact on the formation of free radicals by Aβ. Agents that control ROS may be potentially effective in the treatment of AD given the relationship between Aβdeposition and oxidative stress. For instance, it has been proven that the free radical antioxidant vitamin E can be applied to counteract neurotoxicity caused by Aβ (Chong et al., 2005).

In AD, in addition to this background level of ROS, there are additional more factors:

1. The synthesis of advanced glycation end products (AGE) and OH from H_2_O_2_ are both catalysed by excessive deposits of iron and/or copper.
2. Most senile plaques' surrounding activated microglias(Butterfield et al., 1994) are a source of NO and O2, which can combine to generate peroxynitrite, leaving nitrotyrosine as a recognisablesignature(Castegna et al., 2003).
3. Through peptidyl radicals, Aβ has been directly linked to the production of ROS(Hensley et al., 1994);(Yan et al., 1996).
4. In the presence of transition metals, AGE products may experience redox cycling, which will lead to the generation of ROS. Additionally, AGE products and Aβ activate particular receptors like the Class Aβ scavenger receptor and the Receptor for Advanced Glycation End Products to increase ROS production (Fig. 13) (Davis et al., 1997, Khoury et al., 1996).

**Fig. 12:**
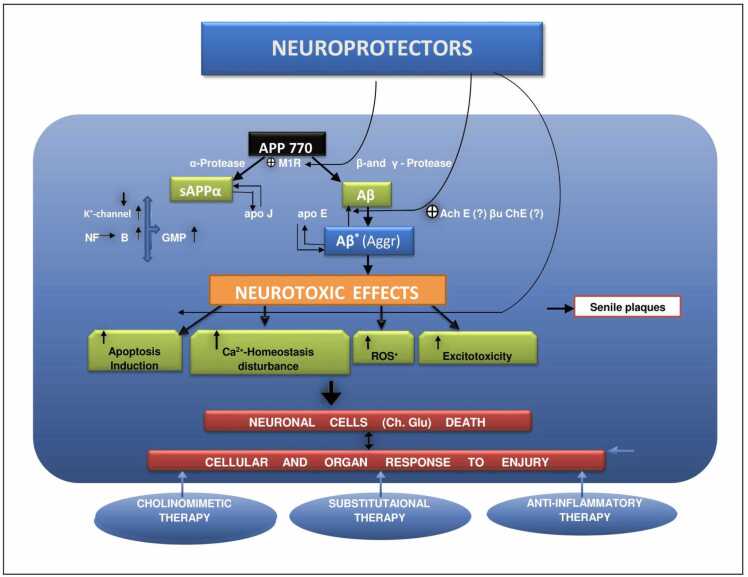
Principal strategic directions for reversing and preventing AP-induced neurodegeneration.

**Fig. 13:**
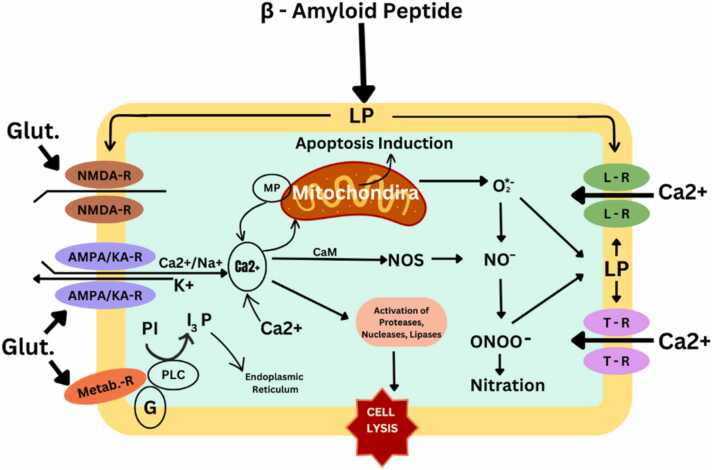
Schematic representation of Aβ's impact on the intracellular calcium homeostasis, ROS production, and cell ageing processes.
5. The mitochondria may be the primary and potentially the initial source of ROS, according to abnormalities in mitochondrial metabolism, such as impairments in essential enzyme activity, which are partly explained by the discovery of the mitochondrial genome(Sensi et al., 1999).
6. Abnormal protein breakdown or proteasomal function (LambonRalph et al., 2003).

In Alzheimer's patients, elevated levels of 8-hydroxy-2'-deoxyguanosine, a sign of oxidative DNA damage, are seen in intact DNA in the cerebral fluid(Arlt et al., 2002); (Keller et al., 1997) However, Alzheimer's patients have significantly lower levels of free 8-OHdG in their cerebrospinal fluid than healthy individuals(Mark et al., 1995);(Mattson and Chan, 2003). This substance is produced during normal cellular repair mechanisms. Additionally, it has been demonstrated that the brains of AD patients have significantly higher levels of the apoptotic marker prostate apoptosis response-4 (Chong et al., 2005).

#### 6.8.1 Generation of reactive oxygen species

In the hippocampus and amygdala of AD patients, Catalase, glutathione peroxidase and reductase, are increased. DNA bases are susceptible to nitrations, protein carbonylations, and hydroxylation-related oxidative stress damage.The brains of AD patients also exhibit reactive oxygen species-induced calcium influx that activates glutamate receptors and causes an excitotoxic reaction that results in cell death(Parks et al., 2001). Reactive oxygen species are produced when oxygen combines with uncontrolled redox-active metals.

#### 6.8.2 Generation of reactive nitrogen species

Through activation of the N-methyl-Daspartateand α-amino-3-hydroxy-5-methyl-4-isoxazolepropionate receptors, glutamate release accompanied by excessive Ca^2+^ influx triggers nitric oxide (NO) synthesis. Aβ stimulates the generation of NO by interacting with glial cells or by NMDA receptor-mediated disruption of Ca^2+^ homeostasis. Peroxynitrite is created when NO reacts with superoxide anion, and the reactive nitrogen species that are produced as a result can significantly increase oxidative stress, resulting in lipid peroxidation, and neuronal death (Arendt, 2001, Masters et al., 2006).

The neuron-specific synthesizing enzymeis aberrantly expressed in the isocortex and entorhinal cortex's potentially susceptible in AD. (Fig. 12) The transcriptional upregulation of nNOS may be a precursor to neurodegeneration.During the S phaseand G1of the cell cycle, cdk2, a crucial regulator of growth arrest, is inhibited by NO, which can cause this under developmental circumstances(Brown et al., 1997).

### 6.9 Inflammations in AD

In addition to astrocytes microglias play a significant function in the inflammatory response in the AD brain. Through the up-regulated expression of phospholipase A2, microglial cells contribute to the chronic inflammatory responses in AD by secreting interleukin-1, producing pro-inflammatory and neurotoxic substances like TNF and IL-1ß, and increasing prostaglandin inflammatory pathway activity(Chong et al., 2005), production of several potentially neurotoxic substances, such as superoxides and glutamate, as well as complement pathway activation and NO (Du et al., 2005, Masters et al., 2006). These pathways produce inflammatory reactions, which may potentially result in brain damage. Membrane attack complexes are created when the complement cascade is activated, and these complexes can phosphorylate the tau protein, which can result in the development of neurofibrillary tangles and cause significant neurodegeneration (Dickey et al., 2006, Ray and Lahiri, 2009).

Aβ may trigger an acute inflammatory response that includes microglial activation, the release of TNFα, and the expression of inducible nitric oxide synthase in neurons as well as the formation of peroxynitrite and neuronal death. It's interesting to note that microglial cells and the perivascular Aβ deposits co-localize in AD patients. It has been noted that microglial activation co-occurs with the development of amyloid plaques(Chong et al., 2005).

A transcription factor called NFκB, which is found in the cytoplasm, controls how cytokine synthesis is carried out. Normally, the inhibitory protein IkB keeps NFκBinactive.NFκBreaches the nucleus after activation and stimulates the transcription of several inflammatory mediators. The chemicals TNFα, Aβ, and secreted APP are among those that can activate NFkB., The regulatory region of the APP, PS, and BACE-1 genes include NFκB sites, according to gene mapping.An enhance in Aβ production ultimately results in the transcription of APP and BACE-1 by NFκB activation (Atwood et al., 1999, Ophir et al., 2005).

### 6.10 Metals and AD

The hippocampus, and cortical vasculature of the brain, which are areas damaged by AD, have high concentrations of these metals, it is likely that Aβ interacts pathologically with redox active metals like zinc, copper, and iron (Barnham et al., 2004); Cuajungco & Fagét, 2003;(Maynard et al., 2005). It may contribute to oxidative stress because they affect hydrogen peroxide (H_2_O_2_) generation (Bush, 2002). Through metal reduction, the binding of trace amounts of these metals to Aβ facilitates the catalytic production ofH_2_O_2_ from oxygen. These metals incompletely understood impact on the oxidative processes underlying Aβ cytotoxicity(De Benedictis et al., 2019); (Newman et al., 2007).

During normal physiology, APP and Aβ interact with Zn, Cu, and to a lesser extent Fe, possibly existing in a delicate balance. Changes in the metabolism of zinc and copper as a result of aberrant Aβ metabolism and synaptic zinc flooding may cause perturbations in this equilibrium and the development of fibrillar Aβ. Thus, this fibril production results in an inflammatory reaction that lowers pH and disturbs zinc homeostasis (Brown et al., 1997, Bush, 2002).

Numerous in vitro studies that show low concentrations of Zn^2+^ promote the quick aggregation of Aβ at physiological pH are consistent with the in vivo studiesAdlard and Bush (Adlard and Bush, 2006); (Gaggelli et al., 2006); (Assaf and Chung, 1984). These findings indicate that the synaptic cleft, where neurotransmission results in peak concentrations of about ∼300 µM Zn^2+^, is an ideal location for Aβ metallation and aggregation(Schlief et al., 2005)and up to 100 µM Cu^2+^(Kardos et al., 2018, Friedlich et al., 2004). The findings of a transgenic mouse model of AD (Tg2576) lacking the zinc transporter 3 (ZnT3) showing a substantial decrease in plaque formation(J.-Y. (Lee et al., 2002); (Linkous et al., 2008), which is responsible for transporting and enriching zinc into pre-synaptic vesicles(Jiang et al., 2007).

The majority of researchers concur that Aβ binds Cu^2+^ and Zn^2+^ in a 1:1 ratio (Clements et al., 1996); (Kozin et al., 2001) Zn^2+^and extra copper causing Cu^2+^has been reported to bind to Aβ in a 2:1 ratio,(Ha et al., 2007, Han et al., 2008). The Aβ metal ions ratio regulates not only Aβ conformation, but also aggregation, according to growing data (Morgan et al., 2002); (Vasudevaraju et al., 2008); (Zatta et al., 2009).

More than just putting Aβ together, copper, iron, and zinc are involved. Additionally, it was discovered that when Cu^2+^ or Fe^3+^bind to Aβ, they become reduced and, through a double electron transfer to O_2_, produce H_2_O_2_. The Met35 residue, which causes Aβ-induced toxicity in cell culture and oxidative stress, may have provided the electron for this reduction. Zn^2+^ appears to play a complex role in the pathophysiology of Aβ. Aβ is precipitated by Zn^2+^ to produce amyloid plaques(De Benedictis et al., 2019); (Barnham et al., 2003).

In the cerebral cortex, the majority of glutamatergic synapses also release Zn along with glutamate, although not all of them do. Due to its capacity to precipitate Aβ quickly and form protease-resistant, "non-structured," aggregates, this cation has been suggested to play a main role in AD. Additionally, studies using animal models of AD have demonstrated that genetically removing synaptic Zn significantly reduces the amount of amyloid plaques, and a number of studies suggest that substances that affect Zn homeostasis can lessen the deposition of Aβ in the brain (Barnham et al., 2003). It has been established that the APP ectodomain and Aβsequence both include putative zinc and copper-binding domains (CuBD). It has been determined that its CuBD has two histidines (His147, 151) that can coordinate Cu^2+^ and reduce it to Cu^+^, as well as the amino acids tyrosine (Tyr168), methionine (Met170) (Bayer and Multhaup, 2005), (Lees et al., 1990).

The pathophysiology of AD has been linked to transferrin, an iron-binding protein, which are important in iron homeostasis and storage. It is possible that transferrin becomes stuck inside plaques when it tries to move iron across cells because it is found in senile plaques rather than its usual site in the cytoplasm of oligodendrocytes. Both the mediator of iron uptake by cells, melanotransferrin and the iron-storage protein ferritin (p97), are changed in AD and are found in and near senile plaques.

Due to the possibility of neurotoxicity, both Cu^2+^ and Zn^2+^(King et al., 2000), the brain possesses effective homeostatic controls and buffers to stop aberrant metal ion compartmentalization.Aβ intriguing target for pharmacological treatments that could help AD is the metal-binding site on Aβ. This strategy uses medicinal chemistry techniques to find substances that prevent the site's harmful H_2_O_2_ production which may be mediated by a different metal-binding site. Similar substances may be helpful in treating cataracts, Parkinson's disease, and ALS, all of which involve aberrant metalloprotein biochemistry(De Benedictis et al., 2019).

For instance, neurofilaments are detected in the NFT and they exhibit phosphorylation dependent changes relatively early in the AD cascade(S. W. (Suh et al., 2000). This multi-subunit protein was purified, and it showed that it binds at least 1copper and 4 zincs in a stoichiometric manner(Phiel et al., 2003). NFs may be assembled in part by metal ions like copper, which encourage the assembly of the light NF subunit.Zinc ions (Malm et al., 2007) and the iron regulatory protein-2, for example, have been found to co-localize with NFT-containing neurons. Zn^2+^ addition produces hyperphosphorylation in mouse neuroblastoma cells (N2a) and human neuroblastoma cells respectively(Björkdahl et al., 2005). As a result of changing the protein's structure and encouraging phosphorylation, ferric and cupric ions can attach to different tau "repeat" motifs(Ma et al., 2005) and inducing its aggregation (Smith et al., 1997, Zhou et al., 2007).

An excessive amount of free radical production caused by redox active transition metals bound to Aβ may have contributed to the increasing synaptic disruption and eventual neuronal death seen in AD(Atwood et al., 1998);(Sayre et al., 2000). Creating an allosterically ordered membrane penetrating oligomer, the addition of Cu or Zn to Aβ can cause a conformational change from β-sheet to α-helix in a lipid membrane environment(Curtain et al., 2001); (Martins et al., 1986); (Miura et al., 2000). The severe oxidative damage connected to Aβ (Arispe et al., 1993)may entail calcium dysregulation, brought on either by membrane calcium channel development or (Frederickson et al., 2005) modulation of an existing channel (Masters et al., 2006).

The aforementioned literature, however, shows that APP and/or Aβ play a significant physiological function in controlling metal-ion levels. The metal theory of AD has been proposed by Bush, Tanzi, and others as a result of this accumulated evidence(Bush and Tanzi, 2008);(Garai et al., 2007), which states that redox-active metal ions can bind to Aβ due to age-related endogenous metal dyshomeostasis in the brain. As a result of Cu^2+^stabilising the neurotoxic, oligomeric Aβ species, this may cause neurotoxicity(Lau et al., 2006), causes the covalent di-tyrosine crosslinking of Aβ and encourages the production of copper-derived diffusible ligands that are resistant to SDS(Barnham et al., 2004);(Beffert and Poirier, 1998). Radicals produced as a result, such as hydrogen peroxide and superoxide, cause oxidative stress damage to DNA, proteins, and lipids, which ultimately results in synapse and neuronal loss (Price et al., 2007).

### 6.11 Apolipoprotein E (ApoE): physiological function

Normal oligodendroglia, astrocytes, and microglia all contain apolipoprotein E, which is crucial for the transportation of cholesterol and phospholipids to areas where neuronal membrane repair and remyelination occur(Y. (Lee et al., 2004); (Masters et al., 2006);Schipper, 2011). Cholesterol and phospholipids are transported by a lipid carrier protein called apoE, which is a component of chylomicrons, LDL, and HDL. Apolipoprotein E is 299 amino acid glycoprotein thought to be crucial for synaptic plasticity and neural repair processes.In the CNS, ApoE may lessen Aβ aggregation and is the most prevalent HDL apolipoprotein.

The brain also contains other apolipoproteins, such as ApoA-I, ApoA-II, ApoA-IV, ApoH, and ApoJ, in addition to ApoE. The potential contributions of ApoA-I and ApoJ to the formation and clearance of A fibrils have been studied. Instead of being made in the brain, ApoA-I is transferred across the BBB. Contrarily, ApoJwhich is expressed in both neurons and astrocytes, is strongly increased after injury. It is known that both ApoA-I and ApoJ bind to A and inhibit its aggregation and toxicity in vitro. The three most commonly seen isoforms of ApoE are ApoE2,ApoE3 and ApoE4 as a result of variations in the coding sequence caused by the three most frequent single-nucleotide polymorphisms. The ε4 allele of the ApoE gene was found to be one of the strongest genetic risk factors for AD, specifically LOAD, when the immunoreactivity of ApoE in amyloid plaques was initially identified(Masters et al., 2006); (Ray and Lahiri, 2009).

The congophilic angiopathy-related A deposition in carriers of the apolipoprotein E4 genotype may significantly contribute to the persistent cerebral hypoperfusion that is frequently seen in AD patients' neuroimaging investigations. It reduces secondary glutamate excitotoxicity in vitro to protect neuronal-glial cell cultures from H_2_O_2_ oxidative stress (Bayer and Multhaup, 2005)and plays a direct and indirect role in the brain's oxidative systems. Aβ and APP interacts directly with the carboxy-terminal domain of ApoE. The development of fibrils prevents the interaction between ApoE and Aβ (Schipper, 2011; Guilloreau et al., 2006).

The cysteine to arginine change at position 112 for isoform 3 and 158 for isoform 2 distinguishes ApoE4 isoform from the other ApoE alleles. The reduced antioxidant impact of the ApoE4 allele may be due to this cysteine, which is thought to be involved in transition metal binding (Saunders et al., 1993).

In 1993, Saunders et al. (Strittmatter et al., 1993) and Strittmatter et al. (Kim et al., 2003) published landmark papers linking the ApoE *ε*4 allele to the development of late-onset, sporadic AD.Depending on the gene dose of the e4 alleles, the presence of the e4 allele raises the risk of AD from 20% to 90% and lowers the age of onset from 84 to 68 years. Additionally, it was discovered that APOEe4 has characteristics of hyperinflammation that cause neuronal damage in the AD brain(Acharya et al., 2002);(Poirier, 2008);(Ray and Lahiri, 2009):

1. The E4 isoform's (Fig. 14) enhanced generation of C-terminal-truncated fragments increases tau hyperphosphorylation and the development of neurofibrillary tangles(Harris et al., 2003);(Sadigh-Eteghad et al., 2015);

**Fig. 14:**
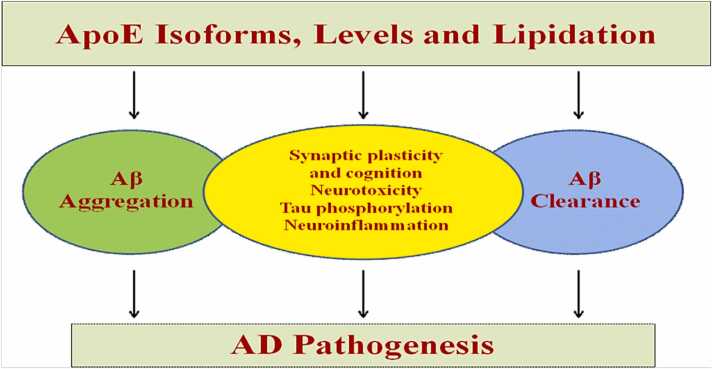
ApoE's pathogenic mechanisms in AD. To comprehend the varied impacts of ApoE isoforms on AD development, a number of pathways have been postulated. According to data, Aβ aggregation and clearance, which affect the time until Aβ deposition begins, are the main ways that ApoE isoforms affect the chance of developing AD.There may be additional mechanisms that contribute to the illness process, including as neurotoxicity, the impact of apoE isoforms on synaptic function, hyperphosphorylation of tau, and neuroinflammation(Poirier, 2008).
2. The neurotoxic peptide deposits more prominently and Aβ removal is less effective in the aged ε4 carriers' brains (Osada et al., 2009); and
3. The severity of dementia in AD is known to be exacerbated by ischemic brain injuries and hypertension-related white matter lesions, and these conditions are more common in patients who test positive for the ε4 protein(Kemp and Bashir, 2001).

### 6.12 Excitotoxicity

In both normal brain function and development glutamate plays a major role. Glutamate neurotransmission disturbances have serious repercussions. Leading to sustained local depolarization of neurons long-term increases in extracellular glutamate tonically activate glutamate receptors, This, in turn, sets off a series of intracellular events that lead to Na+ and Ca2 + influx and further exocytosis of glutamate by activating specific kinases and phosphatases, including calmodulin dependent kinase II (CamK-II), calcineurin, and cyclic AMP) (Palmer et al., 2005, Shankar and Walsh, 2009). Ca2 +influx causes the neuron to die more slowly and activates apoptotic pathways to a lower extent. Excitotoxicity is continuing release of glutamate leads to a spreading of the process(Choi, 1992). The ability of glutamate to be highly toxic while also being essential for neurotransmission strikes a delicate balance between disease and adaptability. A number of brain conditions, including AD, epilepsy, amyotrophic lateral sclerosis and trauma, have been linked to glutamate excitotoxicity. The three types of ionotropic receptors (iGluRs), N-methyl-D-aspartate, kainate, and a-amino-3-hydroxy-5-methyl-4-isoxazolepropionate are activated in order to produce rapid glutamate neurotransmission.

‘‘acute’’ excitotoxicity are recognised by 3 phases:

1. An initial depolarization of the neuronal membrane caused by the AMPA and/or Kainate subtypes of glutamate receptors causes an increase in the inflow of Na+ , Ca2 + , and water molecules into the cell. As a result, the cell experiences what is known as "osmotic swelling" and the magnesium ions that block NMDA receptors dissociate.
2. Hyperactivation of NMDA receptors is followed with a significant increase in the entry of calcium ions into the cell and a several-fold rise in the calcium concentration ([Ca(2þ)]c) in the cytosol. Proteases, nucleases, and lipases are some of the intercellular enzyme systems that are activated as a result, which sets off a series of degenerative events that finally result in cell lysis. The presence of calcium ions is crucial for this phase.
3. Cells undergo exocytosis, which causes a significant release of endogenous glutamate.Phases 1 and 2 of the neurodegenerative process are amplified in the cell as a result of the significant rise in extracellular glutamate concentration and subsequent hyperactivation of glutamate receptors. A "slow" or "metabolic" excitotoxicity is characterised in addition to the "classic" excitotoxicity mechanism. When the cellular energy status is low, this form of neurodegeneration is seen at a normal (not elevated) glutamate concentration. In this case, pathogenic processes are triggered by a disruption of ATP synthesis and mitochondrial activity.

#### 6.12.1 NMDA receptors

L-glutamate and glycine both cause NMDA receptors to become active. These co-agonists must bind in order for the ion channel to fully open. Without membrane depolarization, which needs the preceding activation of AMPA and kainate receptors, extracellular Mg2 + blocks the ion channel. This process is believed to be behind how NMDA receptors contribute to synaptic plasticity and long-term potentiation, which is essential for learning and memory (Walton and Dodd, 2007).

#### 6.12.2 AMPA receptors

GluR1–4 subunits make up the tetrameric complexes that contain AMPA receptors, which are located in post-synaptic densities together with NMDA receptors (Hollmann et al., 1991). Following glutamate binding, AMPA receptors allow an influx of Na+ and K+ . The GluR2 subunit in its edited form, is of particular interest since its inclusion in the AMPA receptor complex results in incredibly low Ca2 + permeability (Park et al., 2016). Without a GluR2 subunit, AMPA receptors are more similar to NMDA receptors in that they permit a large inflow of Ca2 + ions. NMDA-receptor independent LTP is possible with GluR2-deficient AMPA receptor subtypes (Kwak and Weiss, 2006). Ca2 + -permeable AMPA receptors, despite their rarity, may play a role in excitotoxic processes (Kew and Kemp, 2005);(Walton and Dodd, 2007).

#### 6.12.3 Kainate receptors

There are two subfamilies of kainate receptors: GluR5–7 and KA-1 and − 2. Functional receptors can be homomeric, consisting solely of GluR5–7, or heteromeric, combining KA-1 and − 2 with other members of the GluR5–7 subfamily. High-affinity kainate binding sites are created by homomeric KA subunit combinations, not by functioning receptors. The partial binding of ''selective'' antagonists to AMPA receptors, native KA receptor characteristics and location remain elusive (Charpak et al., 1990);(Mulle et al., 2000);(Walton and Dodd, 2007).

#### 6.12.4 Metabotropic glutamate receptors

Based on how well they are connected to intracellular messengers and their pharmacology, the eight metabotropic glutamate receptors can be classified into three classes: group I (mGluR1 and 5), group II (mGluR2 and 3), and group III (mGluR4 and 6–8). mGluRs regulate a number of processes, such as neuronal excitability, synaptic transmission, and neural plasticity depending on their connection with second messengers. Native group I mGluRs induce a gradual activation of voltage-dependent cation influx and decrease K+ conductance in hippocampus pyramidal cells, which contributes to sluggish EPSPs(Bruno et al., 2001); (Chuang et al., 2000);(Congar et al., 1997). By activating the phospholipase C pathway, which results in the production of inositol- 1,4,5-triphosphate and diacylglycerol, these receptors can also cause intracellular Ca2 + release (Vincent et al., 1999).

Recently, research has focused on the mGluR system's protective role in the nervous system. Genomic DNA degradation is stopped by mGluR activation, and in rare situations, it is even reversed (Vincent et al., 1999), modulates endonuclease activation (Bratek et al., 2018), and retains the asymmetry of the cellular membrane(Vincent and Maiese, 2000). To act at or below the level of free radical generation and oxidative stress, cytoprotection by the mGluR system is believed (Ma et al., 2005). According to more recent research, the mGluR protects the vascular system in a similar manner by limiting endothelial cell DNA deterioration, caspase activity, and suppressing a thrombotic state by maintaining membrane asymmetry(Chong et al., 2005).

Other AD-related research show that group I mGluRs can control APP metabolism and hasten the conversion of APP into non-amyloidogenic APP(Chong et al., 2003). Additionally, it has been demonstrated that group III mGluR activation shields neurons from microglial neurotoxicity when Aβ is applied, which may be caused by the control of caspase activity(Hernández et al., 2016);(Chong et al., 2005); (Lin et al., 2001).

#### 6.12.5 Calcium toxicity

There are two stages to the progress of the Aβ's toxicity. First, an early Ca2 + -independent phase that can emerge in a matter of hours, and then a late Ca2 + -dependent phase that can take many days. Blockers of L-type calcium channels are effective at preventing neurotoxicity brought on by the Aβ (25−35) fragment, but not those of N- or P/Q-type channels. The quantity of ROS in cells has an impact on this prevention. It was demonstrated that radical scavengers reduced the buildup of ROS as well as potential-dependent Ca2 + absorption. But specific inhibitors of the voltage-sensitive calcium channel (VSCC) only affect the uptake of Ca2 + (Budvytyte & Valincius, 2023). It's probable that the beginning of LP and buildup of ROS are linked to the early Ca2 + -independent phase of the toxicity of Aβ, which is observed by a decline in the redox activity of intracellular systems. This results in changes to the way the membrane works, such as VSCC depolarization and calcium input into the cell. The latter occurrence depends on calcium and is regarded as the culmination of Aβ's toxicity. These events may be accompanied by variations in the calcium permeability of the glutamate receptor channel complexes. A theory concerning the ability of Aβ to produce new (''exogenous'') calcium-permeable channels and, thus, to cause a calcium-induced cell death, is an alternate theory to the notion of the influence of Aβ on the ''endogenous'' calcium channels. The only supporting data for this process has thus far come from in vitro tests using synthetic bilayered lipid membranes(Bachurin, 2003);(Frederickson et al., 2005); (Sorimachi et al., 1989).

The most notable effect is that Aβ induces Ca2 + to build up, which activates calpains, neutral proteolytic enzymes that are not lysosomal calcium triggered. The ubiquitous calpains 1 and 2 as well as the tissue-specific calpains 3, 8, and 10 and mitochondrial calpain 10 belong to the class of cysteine proteases known as calpains(Siman et al., 1990);(Sorimachi et al., 1993). Under pathological circumstances, calpains can disrupt synaptic transmission, cleave completely soluble and structural proteins in neuronal cells, and harm memory and learning. Changes in these proteases can impact the results of the total APP processing. These proteases, in particular calpain 1, were proposed to be involved in controlled APP processing(Adamec et al., 2002);(Chen and Fernandez, 2005);(Thonberg et al., 2011).

### 6.13 Presenilins, synucleins, notch signalling

The transmembrane protein with eight transmembrane domains is called presenilin (PS). It is expressed throughout the body, especially in the brain, which is enriched in neurons (Defeudis, 2002); Gc & A, 2003). Normal neurogenesis, the development of the axial skeleton, and γ-secretase activity are all impacted by PS1. As a component of the γ-secretase the membranes of the endoplasmic reticulum and Golgi apparatus demonstrated to exist in (Woo et al., 2009), nuclear envelope, cell membrane, and the inner membrane of mitochondria (Chong et al., 2005).Both PS1 (46 kDa) and PS2 (55 kDa), which are membrane proteins with 463 and 448 amino acids, respectively, are widely expressed(Bertram and Tanzi, 2012). 67% amino acid identity are shared by both proteins,PS1 and PS2, (Masters et al., 2006). PS1 enhances the processing and signalling of the Notch1 receptor and binds proteins from the armadillo family, including δ-and β-catenin, suggesting a role in development (Gc & A, 2003; Moir et al., 1999).

Presenilin (PS) mutations boost Aβ-42 synthesis for unknown pathogenic reasons; nevertheless, the growing evidence that PS may be the proteolytic subunit of a γ-secretase complex may offer a preliminary hint. As a result, FAD mutations may directly affect the protease in charge of the final cut that results in Aβ generation, while minute modifications to PS structure may have an impact on the accuracy of the γ-secretase cut. Aβ-42 forms oligomers that are neurotoxic and disrupt neuronal survival and function significantly more quickly than Aβ-40 does. PSs are not solely in charge of the elevated Aβ42 generation linked to FAD. The discovery that PS1 gene ablation in mice causes a significant decrease in both Aβ40 and Aβ42 levels provides yet another direct link between an essential role of PSs in β-secretase activity and all sporadic AD cases (Haass and Steiner, 2002; Kim et al., 2009, Moir et al., 1999).

In the vast majority of familial, EOAD cases, PS1 and PS2 are modified. The exact role that this plays in the emergence of AD is still not fully known. In cultured cells and the brains of transgenic mice, the Aβ42 vs Aβ40 amyloid peptide ratio is thought to increase due to the PS mutations, which are also thought to impair the activity of the γ-secretase complex. The fact that PS1's 6th and 7th transmembrane domains contain two transmembrane aspartate residues that are crucial for the synthesis of Aβ suggests that PS1 is either a crucial cofactor for γ-secretase or could even be γ-secretase itself. Additionally, PS2 has two transmembrane Aspartate residues that seem to be essential for γ-secretase activity(Bulat and Widmann, 2009);(Masters et al., 2006). A γ-secretase complex that includes presenilins, NCT, Aph1, and Pen2 is in charge of cleaving APP and other integral membrane proteins like Notch, E-cadherin, CD44, and others. According to current research, Pen-2 triggers the ultimate maturation of the c-secretase complex, whereas Aph-1 and NCT work together to stabilise the presenilin holoprotein. The stoichiometry of each element in the c-secretase complex has not yet been determined, yet (Artavanis-Tsakonas et al., 1999).

A class of closely similar proteins known as synucleins is particularly prevalent in brain tissue, where pre-synaptic terminals are where they are most enriched. Lewy bodies are mostly made up of α-synuclein, which is particularly abundant in the telencephalon. A core 35-residue fragment of α-synuclein that was first referred to as the non-A-beta component precursor is a significant part of amyloid plaques in AD, making up around 10% of all the proteins there. Both in the cytosol and at certain synaptic locations, α-synuclein appears to be increased in early AD. Alpha-synuclein's physiological purpose is still a mystery (Gc & A, 2003; Bussiere et al., 2003).

Notch is a cell surface glycoprotein that encourages neurite outgrowth and has repeats that resemble EGF. It is typical of many cell adhesion molecules and may only interact with the cell surface in the presence of presenilins. It is a single-pass type I transmembrane receptor that was first identified in the wing-notching mutation-prone Drosophila species(Haleem et al., 2007). In addition to playing a crucial role in neuronal development, the Notch pathway may also play a role in adult brain function. It may regulate synaptic plasticity, long-term memory, neuritic growth, neural stem cell maintenance, proliferation and differentiation in balance, as well as processes such as neurogenesis and neuritic growth that have been linked to cognitive function. Membrane receptor 300 kDa is encoded by the Notch1 gene.Notch-ligand interactions, which govern cell proliferation and differentiation and mediate cell-cell communication, are highly conserved phylogenetically(Haleem et al., 2007).

**Fig. 15:**
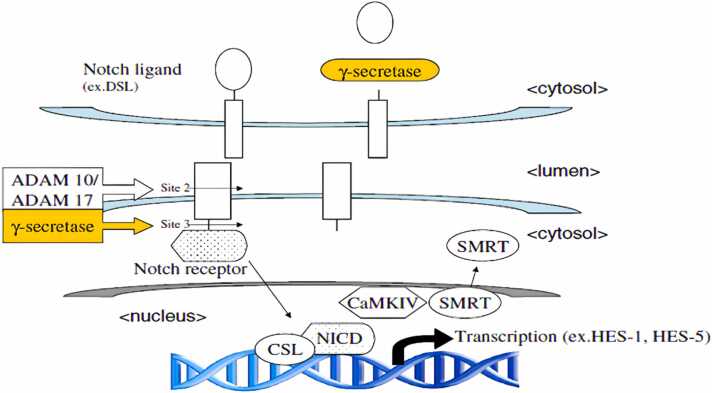
Notch signalling in brain cell-cell communication. The activation of Notch receptors and go through c-secretase-dependent cleavage to produce the Notch intracellular domain when they bind a ligand, such as DSLproduced in a neighbouring cell. NICD moves into the nucleus where it binds to the nuclear cofactor CSL to control replica. It's interesting to note that Notch ligands can also be broken down by presenilin-dependent processes to produce intracellular fragments that are transcriptionally active.

**Notch signalling in AD**(Fig. 15).

In the adult brain, Notch is expressed by neurons, hippocampus where it is most abundant (Donoviel et al., 1999). Notch proteins interact with PSs and APP (Haleem et al., 2007), and play a part in the memory problems linked to AD, in post-mitotic neurons, The phenotype of PS1 deletion mice resembles that of Notch knockout mice (Donoviel et al., 1999)and the phenotype of the PS1/PS2 double knockout is even more comparable (Arumugam et al., 2006), suggesting closely related functions for these proteins. In fact, developmental defects caused by knockouts of any one of numerous γ -secretase components are identical to those generated by Notch 1 and Notch 2 knockouts (Artavanis-Tsakonas et al., 1999).

The Notch intracellular domain is acquired to the nucleus through the intramembranous proteolysis of both APP and Notch, which is mediated by presenilin. The Notch receptor's ability to signal absolutely depends on NICD production. Trans-activating gene expression is necessary for tissue development and renewal, and NICD penetrates the nucleus and does this. A double knockout of PS1 and PS2 completely eradicates the creation of NICDs, and a PS1 deficiency significantly lowers the synthesis of NICDs (Kontush, 2001, McMillan et al., 2018).

In Notch mutant mice with normal acquisition and short-term spatial memory, there were abnormalities in long-term spatial memory.It was recently shown that Notch signalling mediated by γ-secretase affects brain damage (Caricasole et al., 2003). It has been found that the incidence of AD and vascular dementia is significantly enhanced after cerebral ischemia and stroke supports a potential role for Notch signalling in the pathogenesis of AD, according to this finding (Woo et al., 2011).

### 6.14 The Wnt pathway

**Fig. 16:**
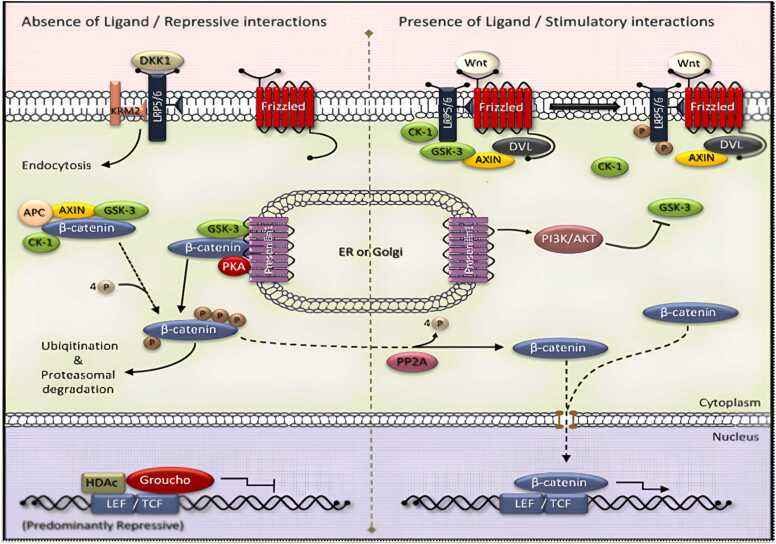
Wnt/b-catenin signalling model simplified. b-catenin is destroyed and its mark genes are primarily inhibited when Wnt ligand is absent (left). The pathway is activated when Wnt binds to its receptor Frizzled and co-receptor LRP5/6. B-catenin degradation is downregulated as a result of DVL's subsequent binding to the intracellular domain of FZ, which causes the recruitment of members of the b-catenin "destruction" complex to the membrane(right).B-catenin is transferred to the nucleus as a result of accumulation, where it controls the transcription of genes that are regulated by LEF/TCF. On the left, repressive interactions are shown. Presenilin increases the phosphorylation of b-catenin, Wnt co-receptor LRP5/6 is internalised as a result of the combined action of DKK1 and KRM2 (Boonen et al., 2009).

Wnt proteins, which take their name from the Drosophila protein “wingless” and the mouse protein “Int-1”, represent a large family of secreted cysteine-rich glycosylated proteins. (Fig. 16)The patterning, differentiation, proliferation, adhesion, orientation, survival, and apoptosis of embryonic cells are all regulated by members of this family of proteins. In order to trigger gene transcription and the release of intracellular calcium (Ca2 +), Wnt attaches to seven-pass transmembrane Frizzled receptors on the cell surface. The canonical route, which is regulated by β-catenin through Wnt-1, Wnt-3a, and Wnt-8. Wnt-4, Wnt-5a, and Wnt-11 are the main components of the non-canonical or Wnt/ Ca2 + pathway, which is another pathway (Chong et al., 2005).

Wnt functions by recruitment of disheveled, the cytoplasmic bridging molecule, to inhibit GSK-3β. By blocking GSK-3β, -catenin is not phosphorylated and is not degraded. While dephosphorylation by protein phosphatases (PP2A), which is possible, stimulates transduction of the Wnt/β-catenin-dependent signal, phosphorylation of β-catenin promotes its ubiquitination and degradation (Boonen et al., 2009). Free β-catenin moves into the nucleus, where it activates lymphocyte enhancer factor (Lef) and T cell factor (Tcf), stimulating the genes that produce the Wntresponse.Wnt-1 inhibits caspase-9 activation pathways via suppressing transcription of -catenin/Tcf, preventing the release of cytochrome c from mitochondria, and other mechanisms. Without Tcf/Lef activity, adenomatous polyposis coli (APC) is therefore allowed to increase the activities of caspases 3, 7, and 9, as well as to break down poly (ADP-ribose) polymerase (PARP), increasing the sensitivity of cells to apoptosis (Chong et al., 2005). Wntsignalling loss may contribute to AD. When protein kinase C activity is increased, which may be regulated by the Wnt pathway, less Aβ can be produced.

**Fig. 17:**
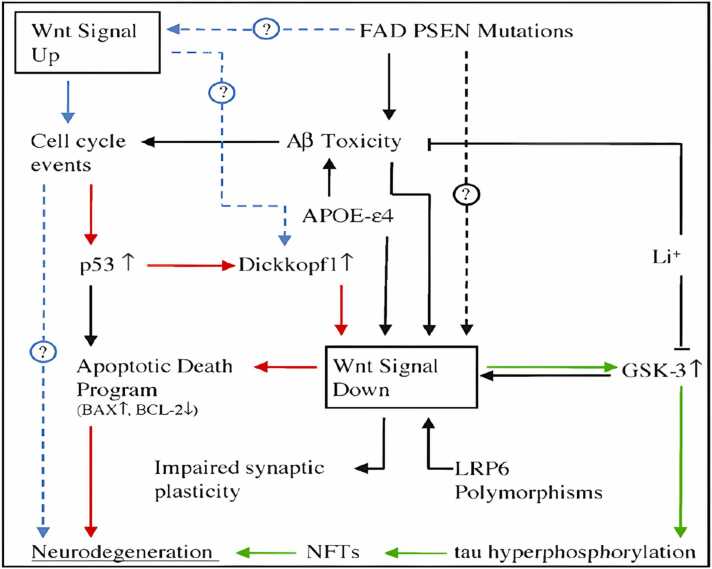
DeregulatedWntsignalling in a pathological cascade linked to neurodegeneration. Both FAD mutations and genetic variants linked to SAD have been demonstrated to deregulate Wntsignalling by both direct and indirect pathways, such as Ab toxicity. The red-hued pathway reflects Wntdown regulation as a secondary effect of abnormally stimulated cell cycle events by an ab, which results in programmed cell death. The possible overactivation of Wntsignalling and its repercussions as a result of FADPSEN mutations are depicted in blue. The potential mechanism that connects tau hyperphosphorylation and related neurodegeneration to the attenuation of the Wnt signal is shown in grey.Other interactions that cause a departure from the typical Wnt/b-catenin signal are shown in black. When a pathway is denoted by a question mark, it means that the relationship between the two is either unclear or that there are conflicting findings regarding the interaction: Li+ , lithium; NFTs, neurofibrillary tangles(Boonen et al., 2009).

**Fig. 18:**
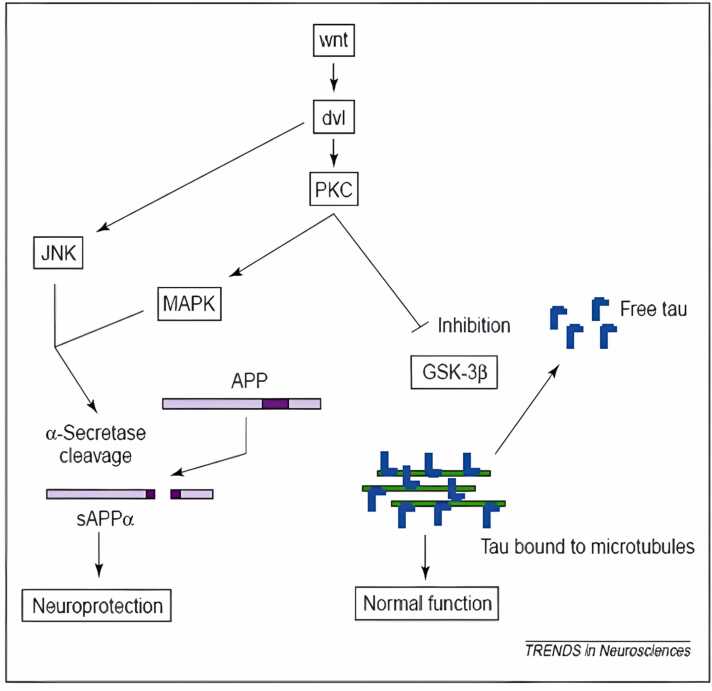
The idea of wntsignalling,Wnt transmits a signal via protein kinase C and dvl. Dvl and wnt promote the production of secreted amyloid precursor protein (sAPP)α and prevent tau phosphorylation by glycogen synthase kinase-3. Both of these processes may be necessary and typical. It is believed that the loss of the wnt signal would lead to changes in tau phosphorylation and sAPP levels, two clinical symptoms AD. (O’Brien & (O'Brien and Wong, 2011).

It has been demonstrated that PS1 inhibits Wntsignalling and interacts with β-catenin to increase its turnover. Dishevelled, a well-known downstream Wntsignalling pathway (Fig. 17, Fig. 18) transducer, can also control APP's α-secretase cleavage through PKC/MAPK dependent pathways, resulting in an increase in the synthesis of soluble APP (sAPP). By producing more sAPP and lowering tau phosphorylation, dishevelled enhance neuronal protection during neurodegenerative diseases. Modulation of the Wnt pathway and its elements may therefore provide innovative therapeutic strategies to combat neurodegeneration in AD (Chong et al., 2005).

Alteration of Wnt pathway elements could be a key factor connecting plaque and tangle pathology in AD(Mudher and Lovestone, 2002). The amyloid theory states that tau tangle development happens after Aβ pathology (Hardy and Selkoe, 2002), positioning suppression of the Wnt cascade after Aβ but before tau. As a significant inhibitor of GSK-3 activity, the Wnt pathway has been linked to tau disease. Inhibition of the Wnt signal causes GSK-3 levels to rise, which causes MAP-tau to become hyperphosphorylated and assemble into tangles (Alzheimer’s association update, 2011, Bjorkdahl et al., 2005, Boonen et al., 2009, Chiu et al., 2013).

### 6.15 Protein kinase B (Akt)

When Akt is active, it can offer defence against cellular harm. Two significant residues, Thr308 and Ser473, are thought to be required for the activation of Akt.To provide defence against genomic DNA deterioration and membrane phosphatidyl serine exposure, Akt is phosphorylated by phosphoinositide-dependent kinase 1. Early research has shown that excessive Akt expression in cerebellar granule neurons inhibits apoptosis in response to growth factor removal. Primary hippocampal neurons, cerebral vascular endothelial cellsexhibit increased cell survival when subjected to free radicals due to Akt. The injection of anti-Fas antibodies, DNA damage, oxidative stress, matrix separation, and transforming growth factor-β application are a few more toxic insults that increased Akt activity can help cells survive. In both neurons and ECs, increased Akt activity can stop the externalisation of cellular membrane phosphatidyl serine in response to a range of stressors that include anoxia, exposure to free radicals, and oxygen-glucose deprivation. In order to stop microglial activation, Akt also seems to control membrane PS externalisation (Chong et al., 2005).

**GSK-3β and bad include the downstream substrates of Akt**.

In CNS Akt controls the activity of GSK-3β, a serine/ threonine kinase. Although Akt's phosphorylation of GSK-3β at Ser9 causes the enzyme to become inactive, it's significant to note that phosphorylation of GSK-3β at Thr216 causes the enzyme to become more active, which can happen during neuronal degeneration. It's interesting to note that GSK-3β has been linked to the neurotoxicity of Aβ during AD (Cuajungco and Faget, 2003).

Numerous research have suggested that the Bcl-2 family members' regulation is different in the AD brain.It has been shown that proapoptotic and antiapoptotic proteins are both present in greater amounts in AD brain. Those in the Bcl-2 family. By phosphorylating the serine residues in Bad, a pro-apoptotic Bcl-2 family member, Akt renders it inactive. Bcl-2 proteins, of which Bad is a member of the Bcl-2 homology 3 subfamily, are involved in the control of apoptosis (Barnham et al., 2004, Chong et al., 2003).

### 6.16 Caspase (Chong et al., 2005)

Caspase processing affect the microtubule-associated protein tau, which is involved in the creation of intracellular neurofibrillary tangles. Caspases 1, 3, 7, and 8 can cleave tau at Asp421, producing a shortened tau protein (1−421) that assembles more easily into tau filaments. The polymerization of tau protein that has been shortened (1−421) is prevented by the C-terminal peptide (422−441) that is created by caspase cleavage at Asp421 in tau. By cleaving tau's C-terminal peptide, a regulatory inhibitor, by caspase, tau's polymerization is accelerated and NFT is created. Tau, a protein associated with neuronal microtubules, is essential for the development of neuronal architecture.

Microtubules are unable to link with a soluble, dephosphorylated tau fragment of 17 kDa, and it is formed after tau breakage in AD. Caspase inhibitors prevent the production of this fragment, hence apoptotic damage might not happen without its accumulation in cells. Caspase cleavage of tau is results in the formation of truncated tau protein which are involved in the pathology of AD. An increase in tau degradation was seen in frontotemporal dementia cases, which was histologically linked to DNA fragmentation and caspase-3 activation in neurons. These findings imply that tau cleavage may play a substantial role in the pathophysiology of AD when combined with the biochemical information on how caspase breaks down tau (Kaminsky et al., 2005).

There has also been speculation that the progression of AD may be aided by the cleavage of presenilins by caspases. A number of research conducted about ten years ago revealed that human brain tissue and cells both contain caspases, which can also cleave APP. In vitro, caspase-3 cleaves APP at a number of locations, two of which are in the luminal portion of the protein and one of which is in the cytoplasmic tail of the protein. (Bulat and Widmann, 2009, Cacabelos et al., 2003).

### 6.17 Vascular dysfunction as risk factors for AD

Type 2 diabetes and cardiovascular disease are risk factors for AD. The synthesis of Aβ is thought to be promoted by vascular risk factors such hypertension and high cholesterol (Acharya et al., 2002), even though being overweight is now linked to AD (Waddington, 2012). The E4 allele is associated with an increased risk of developing AD and vascular dysfunction because it seems to enhance vascular risk factors (Acharya et al., 2002). Balakrishnan et al.'s latest study looked into whether obesity affects the amounts of the amino acid Aβ through influencing AD development (Ray and Lahiri, 2009). This is the first study to demonstrate that being overweight or obese raises plasma levels of Aβ-42, which increases the risk of developing AD. Recent research further suggests that neurovascular impairment is a key aspect of persistent AD neurodegeneration. According to the neurovascular hypothesis put out by Zlokovic et al., there is improper clearance of Aβ across the BBB. In one case, poor clearance, probably as a result of aberrant angiogenesis, leads to elevated Aβ levels. Additionally, a different route might entail elevated Aβ concentrations along with atherosclerosis. These mechanisms are thought to work together to prematurely age the cerebrovascular system. The BBB is compromised, there is an ionic imbalance and metabolic poisoning in the brain interstitial fluid, and these factors eventually cause synaptic dysfunction and neuronal death and damage, which are clear diseases in AD(Harris et al., 2003);(Reddy, 2013).

### 6.18 Genetic and epigenetic approach towards AD

Conrad Waddington introduced the word "epigenetics" in 1942 and described it as "the branch of biology which studies the causal interactions between genes and their products, which bring about the phenotype" (MacDonald and Roskams, 2009, Schumacher and Petronis, 2006) (MacDonald and Roskams, 2009); (Schumacher and Petronis, 2006). It refers to the study of reversible modifications to gene activity that take place without a change in the DNA sequence and are mostly mediated by adjustments to DNA methylation and chromatin structure(Reichenberg et al., 2009). Epigenetic mechanisms, which alter the DNA's structure and related histones chemically, can affect gene transcription and may be extremely important in the interaction between genetic and environmental variables in creating a subject's phenotype (Chouliaras et al., 2010),(Jaenisch and Bird, 2003).

**Table 1:** AD risk genes.

Familial genes	Function in Alzheimer’s disease	Cause on Alzheimer’s disease
APP	It is cleaved by secretases which results in both the generation of Aβ and non-amyloidogenic byproducts.	increased production of Aβ42
PSEN1	It is a part of the enzyme secretase, which is involved in the conversion of APP to Aβ.	increased production of Aβ42
PSEN2	Participates in APP conversion within the α-secretase complex.	increased production of Aβ42
SorL1	Regulates APP trafficking and reduces Aβ production when overexpressed. Lower SorL1 levels observed in Alzheimer's patients.	NA
APOE	Affects Aβ transport and clearance. APOE4 alleles increase Alzheimer's risk 3–10 times and are associated with cholinergic dysfunction and higher amyloid load.	3–10 times increased
GSK3β	Phosphorylates tau, leading to tau tangle formation. Activated by APP cleavage byproducts and PSEN complexes	1·7 times increased.
DYRK1A	Involved in tau and APP phosphorylation, increasing amyloidogenic processing.	T allele is less frequent in people with AD.
Tau	In NFTs, tau is hyperphosphorylated. Depending on the presence of the N-terminal exons 2 and 3 and the exon 10 microtubule binding domain, tau can be spliced into six different isoforms. Tau mutations can have an impact on microtubule binding efficiency and splicing. The tau haplotype influences the amount of tau splice isoforms expressed and is linked to AD.	H1C haplotype more frequent in AD. No Alzgene meta-analysis ofthe haplotype21,22
PICALM	Associated with enlarged endosomes in early-stage AD	Alzgene odds ratio of 0·87 for rs541458

Epigenetic processes can cause long-term changes in cellular function by affecting the structure of chromatin, gene transcription, and gene expression(Hsieh and Eisch, 2010). Some Studies revealed that aberrant epigenetic patterns may be acquired throughout developmental stages and DNA methylation, histone acetylation, collectively known as epigenetics, in the neuron may vary with ageing. This may explain the reported anomalies in AD (Wu et al., 2010).DNA methylation and histone modification are the most frequently researched epigenetic processes (Jaenisch and Bird, 2003, Mehler, 2008).

#### 6.18.1 DNA methylation

CpG units, where a cytosine nucleotide occurs adjacent to a guanine nucleotide in the linear sequence of bases, are added a methyl group by S-adenosyl methionine, especially close to promoter regions of genes (Foley et al., 2009); Gräff & Mansuy, 2008). Typically, DNA methylation inhibits transcription. In or near promoter regions, CpG units are frequently tightly clustered, forming so-called CpG islands. In general, the dependence of transcription level on DNA methylation increases with the number of CpG islands present in the promoter of a gene (Bird, 2002), (Jaenisch and Bird, 2003). Multiple processes in cells, such as the DNA methylation is necessary for the maintenance of X chromosome inactivation, tissue-specific gene expression, genomic imprinting, silence of repetitive and centromeric sequences, cancer, and ageing. Mechanistically, a methylated cytosine can inhibit or encourage the recruitment of regulatory proteins, which can regulate transcription (Eugene et al., 2016); (Schumacher and Petronis, 2006).

DNA methyltransferases are enzymes that methylate DNA. Recent research has revealed that the methylation potential, also known as the ratio of SAM to SAH, which is closely tied to the one-carbon metabolism, is necessary for DNA methylation. SAM is transformed into SAH by methyltransferases, which modify their target molecules by adding methylcytosine (Jaenisch and Bird, 2003).

#### 6.18.2 Histone modifications (Jaenisch and Bird, 2003)

The chromatin state can also influence how genes are expressed. DNA is bundled in nucleosomes, which are formed by 147 DNA base pairs encircling a histone (H) octamer. Chromatin is made up of DNA-protein complexes. 2 copies of each H2A, H2B, H3, and H4 are used to construct a histone octamer(Richmond and Davey, 2003); (Felsenfeld and Groudine, 2003). Either euchromatin, a decondensed, active arrangement of chromatin, or heterochromatin, a condensed, inactive condition of chromatin, is possible. The covalent modification of histones at specific amino acid residues on their amino (N) terminal tails is the most well-studied method of chromatin remodelling in the brain (Berger, 2007). These alterations include lysine (K) residue acetylation, ubiquitylation, or SUMOylation, arginine (R) residue methylation, and serine (S) or threonine (T) residue phosphorylation (Bannister and Kouzarides, 2005).

Co-activators of transcription, such cyclic adenosine monophosphate response element binding proteinhave endogenous HAT activity that alters the structure of chromatin, thereby typically stimulating gene transcription. The HDACs can counteract the actions of HATs by removing acetyl groups from K residues in the amino-terminal tails of other proteins and core histones, so typically inhibiting transcription (Atwood et al., 2000a, Brown and Bal-Price, 2003).

Histone methyltransferases have the ability to methylate histones on either K- or R-residues, causing changes in the structure of the chromatin that can be either condensing or relaxing. The chromatin structure may be affected by methylation by generating binding sites for additional regulator proteins. While R-residues can be either mono- or dimethylated, while K can be mono- or diortri-methylated. Since there are 24 sites for histone methylation on each histone tail, there are a tonne of different ways to combine these sites to determine the methylation status of a particular histone (Chuang, 2004, Robles, 2009).

## 7 Drug therapy

The following three major objectives should be pursued in the treatment of disorders of adult-onset dementia, including AD: (a) improving cognitive performance, (b) reining in problematic behaviour, (c) delaying the onset of disease are all examples of positive effects(X.-X. (Zhang et al., 2021).

**The evolution of AD has some difficulties for the development of drugs** (Barrow, 2002).

1. The shortage of AD markers in pre – clinical stage
2. Deficiency of markers for AD phenotype
3. The challenge of finding compounds that act just on sick cells
4. Because most medications only influence a small portion of the etiopathogenic chain, they have minimal effects.
5. Need of a prolonged period of observation to prove delay in progression
6. Work with large human sample sizes to produce clinical proof and statistical significance
7. High expense of trials intended to determine changes in AD evolution (Graff and Mansuy, 2008)

For the treatment of AD, Statins, PPARγ agonists, NSAID's, neurotrophic molecules, and even metabolic or nutritional drinks are among the more than 50 substances currently being studied in various stages of clinical trials. Additionally, there are numerous additional candidate molecules that are currently undergoing pre-clinical testing and are anticipated to enter clinical trials.The Food and Drug Administration recently granted Memantine (NamendaR) approval for the treatment of AD in addition to donepezil (AriceptR), rivastigmine (ExelonR), and galantamine (ReminylR).Therapy is still limited to symptomatic palliative measures, as there has not yet been any evidence that any treatment may stop or reverse the underlying illness process (Biran et al., 2009); (Masters et al., 2006).The two anti-Aβ monoclonal antibodies, Aducanumab and Lecanemab, that were approved by the FDA in 2021 (Catarina et al., 2010).

**Fig. 19:**
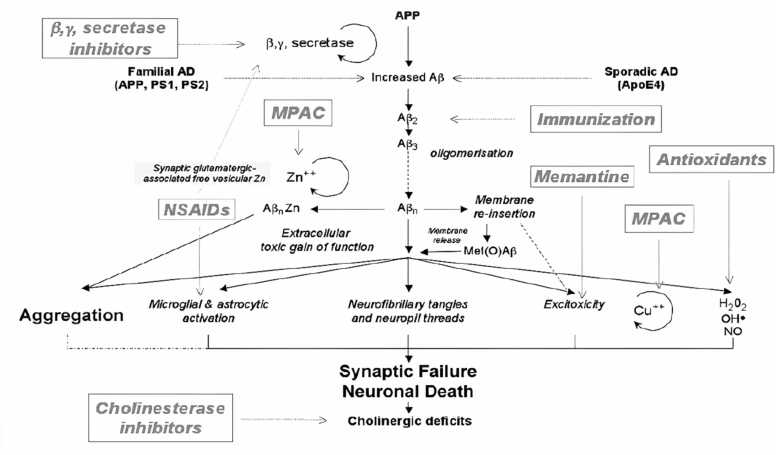
Illustrations of the pathophysiology of AD and the role of Aβ, as well as conventional and cutting-edge therapy approaches. Raise in production or fall in clearance of Aβ causes aggregation, and neuronal injury through a number of neurotoxic mechanisms, including the production of oxygen and nitrogen radicals interactions with transition metal ions, hyperphosphorylation of tau to form neurofibrillary tangles, inflammatory response via microglia, and astrocytic activation resulting in synaptic dysfunction and cell death.The target(s) are indicated by the dotted arrows, which are bolded in grey and boxed with the therapeutic actions. APP, AD, and metal-protein attenuating compound (Masters et al., 2006).

The "amyloid cascade hypothesis," which has dominated the AD field for the past 20 years, has served as the foundation for most of these pharmacological drugs' design and/or development.According to this idea, the primary cause of AD is the metabolism of the Aβ-peptide which, along with the subsequent accumulation of τ-protein aggregates, causes neuronal, synaptic dysfunction, loss of microglial activation and neuronal death. Since both of the main cerebral proteins implicated in the pathophysiology of AD as shown in (Fig. 19), τ and Aβ, are being targeted by the majority of the pharmacological treatments under development. We shall give a general summary of the therapy modalities being created at present time to treat AD in this review (Mangialasche et al., 2010).

**Therapeutic strategies for AD (**(Davies and Koppel, 2009)**;** (Sorimachi et al., 1989)**;** (Woo et al., 2011)**)**.

1. Inhibit β-, γ-secretase in order to stop the development of amyloidogenicAβ
2. Aiming at Aβ's oligomers to stop Aβ's oligomerization or speed up its removal from the cerebral cortex:
3. Immunotherapy: Vaccination with Aβ
4. Selective degradation &Destabilizationof Aβ oligomers:
5. Anti-inflammatory strategy
6. Changing the homeostasis of cholesterol
7. Use a comprehensive amyloid-based approach to avoid synaptotoxic and neurodegenerative effects

Therapeutic approaches for Alzheimer's disease (AD) target various aspects of the disease, focusing on key proteins involved, including amyloid-beta (Aβ) and tau. Below are different therapeutic strategies categorized by their protein targets (Schipper, 2009):

**Aβ-targeted therapies**.

**Inhibition of β- and γ-secretase**.

These therapies aim to halt the production of amyloidogenic Aβ peptides by inhibiting the enzymes responsible for their cleavage.

**Oligomer disruption and clearance**.

Strategies focus on preventing the oligomerization of Aβ or enhancing its removal from the cerebral cortex, thereby reducing toxic Aβ oligomers.

**Immunotherapy**.

Vaccination approaches involving Aβ immunization seek to stimulate the immune system to recognize and clear Aβ aggregates [154, 278, 27].

**Selective degradation & destabilization of Aβ oligomers**.

Therapies aim to selectively target and break down Aβ oligomers, mitigating their neurotoxic effects.

**Tau-targeted therapies**.

**Anti-tau strategies**.

Therapeutic interventions specifically designed to target tau protein abnormalities and aggregation are under investigation. These therapies aim to prevent or slow down tau-related neurodegeneration.

**Multimodal approaches**.

**Anti-inflammatory strategies**.

Inflammation plays a role in AD progression, and anti-inflammatory therapies aim to mitigate neuroinflammation as part of a comprehensive treatment approach.

**Cholesterol homeostasis modification**.

Strategies focused on altering cholesterol homeostasis in the brain to potentially impact AD progression [27].

**Comprehensive amyloid-based approach**.

A holistic approach addressing Aβ pathology to prevent synaptotoxic and neurodegenerative effects, which may involve combination therapies targeting various aspects of Aβ metabolism [154].

These therapeutic strategies represent ongoing research efforts to develop effective treatments for Alzheimer's disease, addressing both Aβ and tau pathologies. While some are in advanced stages of development, others remain areas of active investigation.

### 7.1 Drugs to prevent Aβ aggregation and clearance (Greco et al., 2009)

Chemical heterogeneity and unclear pharmacodynamics characterise non-peptidic anti-aggregants investigated to date. Anti-aggregants may function by attaching to Aβ monomers, blocking oligomerization and facilitating elimination; Substances with high CNS bioavailability, low immunogenicity, and low toxicity are diificult to find (RimaBudvytyte and GintarasValincius, 2023).

Affitopes, which are short peptides that imitate some aspects of native Aβ1–42 but lack its sequence identity, are the basis of the active immunisation technique. The affitopes AD-01 and AD-02 both target the N-terminal Aβ fragment and exhibit disease-modifying characteristics in animal models of AD. Active immunotherapy ensures consistently high antibody concentrations, necessitates minimal follow-up visits, and is less expensive.

Monoclonal antibodies that target Aβ are the foundation of passive immunotherapy, which aims to accelerate Aβ's clearance. Results from animal research have demonstrated that anti-Aβ antibodies can inhibit the development of oligomers, lower brain amyloid load, and enhance cognitive functioning. Bapineuzumab (AAB-001), solanezumab (LY-2062430), and MABT-5102A are the several monoclonal antibodies, generally given intravenously, are being tested in patients with AD.Passive immunotherapy makes it easier to target particular Aβ epitopes and allows for quicker management of antibody titres. In older adults, who have decreased vaccine responsiveness, passive immunisation may be more beneficial than active immunotherapy.

The finding that leptin, a pluripotent peptide produced by adipocytes that lowers Aβ levels and attenuates tau phosphorylation, suggests yet another strategy to lower Aβ production (Tezapsidis et al., 2009), and improves cognitive function (Lieb et al., 2009) in animal models. Higher leptin levels in people are linked to larger brain volumes, less brain atrophy, and a lower incidence of dementia (Gustafson, 2006); (Kurz and Perneczky, 2011).Currently no clinical trials on leptin in AD are being conducted Aisen et al., (Aisen et al., 2008).

Tramiprosatepreferentially binds to soluble Aβ-peptide, prevents amyloid from forming sheets, keeps Aβ from fibrillating, and so prevents the production and deposition of amyloid (Wilcock et al., 2008). Patients with mild dementia had a decreased rate of decline on activities of daily living and on global rating, according to a phase II research (Gauthier et al., 2009). A phase III trial, however, failed to confirm these findings (Hawkes et al., 2010).Additionally, the auto-aggregation of soluble Aβ fragments is inhibited by the stereo-isomeric form of inositol known as scyllo-inositol (ELN D005). It lessens brain amyloid burden and restores memory impairments in transgenic mice (Desire et al., 2008; Van Tijn et al., 2008).

### 7.2 α-secretase inhibitors

testosterone, oestrogens, Statins, and protein kinase C activators are other medications that have been studied in clinical trials and have the ability to enhance α-secretase activity. However, there isn't any proof right now that they're used in AD. In phase 2 clinical trials, etazolate (EHT 0202) is a α-secretase stimulator that shifts APP processing to the non-amyloidogenic route(Landreth, 2007); Aisen et al., (Aisen et al., 2008).

### 7.3 β–secretase inhibitors

Oral medications for type 2 diabetes called thiazolidinediones, such as rosiglitazone and pioglitazone, work as β-secretase inhibitors by activating the nuclear peroxisome proliferator-activated receptor.By boosting APP's ubiquitination, activation of these receptors can inhibit the production of β-secretase and accelerate its destruction.Although it's unclear whether rosiglitazone can enter the human CNS, pioglitazone can cross the BBB (Henley et al., 2009). PPARγ agonists' therapeutic effects on AD may result from how they affect insulin activity, lipid and carbohydrate metabolism, and inflammation. The neuropathology of AD appears to be facilitated by insulin resistance and peripheral hyperinsulinemia. Rosiglitazone and pioglitazone improve peripheral insulin sensitivity while lowering insulin concentrations, which compete with Aβ for the insulin-degrading enzyme's concentration (Bolognesi et al., 2009); Aisen et al., (Aisen et al., 2008).

### 7.4 γ–Secretase inhibitors

Aβ secretase inhibitor called semagacestat (LY450139), which decreases CSF Aβ and seems to be well tolerated, is currently undergoing phase 3 clinical studies (Schneider et al., 2005). Other γ secretase inhibitors, such as MK0752 in phase 1 and BMS-708163 in phase 2, which has a relatively low risk of Notch-related side effects, are in early clinical trialsAisen et al., (Aisen et al., 2008).

### 7.5 Therapies targeting tau

The amyloid cascade theory views alterations in tau that result in NFT production as secondary processes, and as a result, this area has mostly been neglected in terms of treatments. The identification of mutations in the human tau gene, which led to the neurodegenerative conditions commonly known as frontotemporal dementia or tauopathies, altered perception.The notion that the alterations in tau found in AD may be responsible for cell death even if they are secondary to Aβ toxicity stems from the observation that even relatively minor changes in tau can trigger neuronal death (Schenk et al., 2006). Currently, there are two main strategies in this field (Schenk et al., 2006):

#### 7.5.1 Prevention of tau aggregation

Numerous studies on tau aggregation have demonstrated that nonphosphorylated tau can form filamentous aggregates, and they have used these systems to search for potential tau aggregation inhibitors. To promote tau aggregation, it include polyanions like heparin, RNA, or arachidonic acid(Schenk et al., 2006); (Davies and Koppel, 2009).

#### 7.5.2 Prevention of tau phosphorylation

There is little disagreement that tau is hyperphosphorylated in the elderly human and/or animal brain, on average it has an approximately 2–3 moles of phosphate per mole of proteinwhile tau isolated from the Alzheimer brain contains 6–8 moles of phosphate per mole of protein. With the underlying premise that improper activation of a protein kinase activity causes this rise in tau phosphorylation, the overwhelming majority of applied research on tau has focused on the development of drugs to prevent this. Formally, an aberrant kinase activity or a protein phosphatase deficit could be the culprits, but it is typically simpler to create enzyme inhibitors than enzyme activators. Numerous protein kinases may have been activated as a result of the increased phosphorylation at specific locations on tau, according to careful study of these sites. This conclusion is based on research that identified both phosphorylation at sites without/with a proline residue following a serine or threonine, which is necessary for the action of "proline-directed kinases."The likelihood of a cascade of kinase activity has increased, and it has been extremely challenging to pinpoint a single crucial kinase that is responsible for turning tau into NFT(Masters et al., 2006); Gómez-Ramos et al., 2004; (Sensi et al., 1999). Tau molecules are prepared for further phosphorylation by GSK3β by DYRK1A, and this protein may also play a role in the interaction between tau and Aβ(Ballard et al., 2011).

Tau has a significant number of possible phosphorylation sites, which is a key feature. Kinases that phosphorylate tau have 2 groups (Metcalfe and Figueiredo-Pereira, 2010);(Selenica et al., 2007):

1. Protein kinases that are proline-directed and phosphorylate Ser-Pro or Thr-Pro tau motifs. These include cyclin-dependent kinase 5, glycogen synthase kinase-3β, and stress kinases including c-Jun N-terminal kinase and p38.
2. Protein kinases that do not phosphorylate proline-followed serine or threonine residues. Some of them are calcium calmodulin-dependent kinase II, protein kinases A,B, C.
3. It has also been demonstrated that several other kinase inhibitors, such as SRN-003–55, CHIR-98014, and SB216763, lower phosphorylated tau levels in AD mice models (Kuret et al., 2005). A humanised monoclonal antibody called Bapineuzumab (AAB-001) is directed against the N-terminus of Aβ. A monoclonal antibody called solanezumab (LY2062430) binds selectively to soluble Aβ and alters its tendency for auto-aggregation. PF-04360365 and Gantenerumab (R1450), two other monoclonal antibodies, are in phases 1 and 2, respectively Aisen et al., (Aisen et al., 2008).

#### 7.5.3 Inhibitors of fibrillization

Nucleation and extension are the first two processes in the development of an NFT. Small compounds may be able to inhibit either process, although the kinetics is frequently complicated and inhibition is frequently lost as inhibitor concentration rises. The rate-limiting step for fibrillization in the case of tau is nucleation, which can be exponentially increased by adding a nucleation inducer (anionic surfactant). However, tau phosphorylation improves the efficiency of the extension stage (Trojanowski et al., 2005). By raising the critical concentration required for the filament to extend into polymers, small antagonists of fibrillization can exert their action. Important considerations to keep in mind include the possibility that tau phosphorylation can be seen in overexpression systems, could overcome this inhibition. The inhibitor/tau interaction may also be complex, and the inhibitor dosage response curves, which show reduced efficacy at rising inhibitor concentrations, may be explained by inhibitor dimerization ormultimerization (Schenk et al., 2006; Bleakman et al., 2002).

#### 7.5.4 Microtubule stabilizing agents

**Fig. 20:**
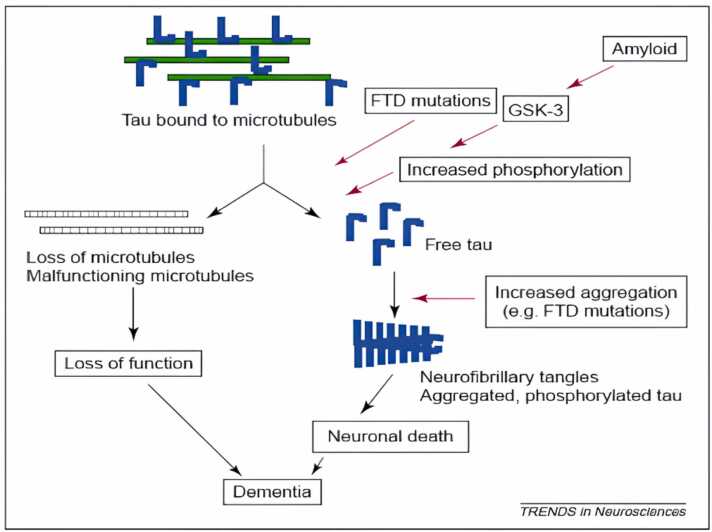
The tau and tangle hypothesis: Phosphorylation, mutations that change function directly, & isoform expression all prevent tau from attaching to microtubules.Reduced tau binding to microtubules could lead to more free tau, which, under the right circumstances, will self-assemble to create paired helical filaments that are insoluble.Tau aggregation without mutations may be caused by elevated phosphorylation, protease activity, or exhibition to polyanions such glycosaminoglycans. The exact mechanism is unknown. (Mudher and Lovestone, 2002).

By imitating tau, (Fig. 20) a protein that acts as a glue to hold tubulin monomers together, stabilising drugs like paclitaxel may aid in maintaining microtubule integrity. Although many microtubule stabilisers have been found, they are incredibly rare and frequently isolated from natural sources. Programmes are being carried out to locate substances with the optimum pharmacokinetic properties by screening derivatives of well-known microtubule stabilising agents (Ballatore et al., 2005); (Schenk et al., 2006); F. (Zhang et al., 2003).

### 7.6 Tau kinase inhibitors

#### 7.6.1 GSK-3 inhibitors

GSK-3 inhibitors can be divided into two categories: those that interact directly with GSK-3 and those that indirectly raise GSK-3's N-terminal phosphorylation. By causing N-terminal phosphorylation of GSK-3, in addition to inhibit GSK-3 directly, lithium (Li+) and other GSK-3 inhibitors have also been shown to inhibit GSK-3 indirectly (Huang et al., 2006), either through activation of Akt or inhibition of protein phosphatase-1 (Beaulieu et al., 2008); Bussière et al., 2003). It has been demonstrated that Li+ , a non-specific GSK-3 inhibitor used in the treatment of bipolar disorders, stimulates the Wnt pathway. Recent research indicates that -arrestin 2 (βARR2) is necessary for the therapeutic activity of Li+ , even if the precise molecular pathways of Li+ action and its pleiotropic event have not yet been fully understood (Behl and Moosmann, 2002). βARR2 participates in the development of signalling complexes that enable independent signal transmission through G-protein coupled receptors. In animals with βARR2 knockout, phosphatidylinositol 3-kinase/AKT pathway activation prevents Li+ from inhibiting GSK-3. According to Beaulieu and colleagues, Li+ disturbs the AKTβ-ARR2-PP2A signalling complex, which typically inactivates AKT, causing AKT to become activated and GSK-3 to become inhibited (Boonen et al., 2009).

**Fig. 21:**
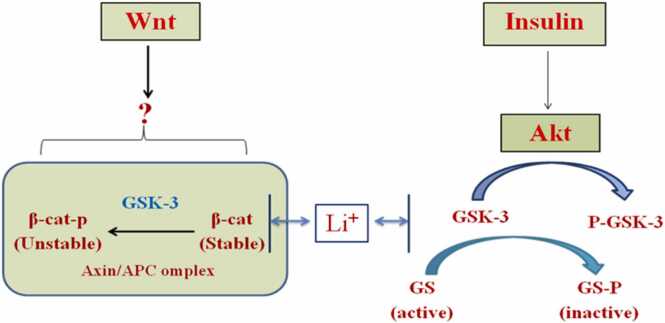
The function of GSK-3 in insulin and Wntsignalling When the Wnt or insulin pathways are activated ligand-dependently, Glycogen Synthase Kinase-3 is inhibited by cause of it antagonises certain branches of these pathways. By phosphorylating GSK-3's N-terminus (P-GSK-3: phosphorylated GSK-3), Akt is activated by insulin and inhibits GSK-3 as a result. Through a mechanism that is unidentified and separate from N-terminal phosphorylation, wntsignalling inhibits GSK-3 linked to the axin complex. In order to enable GSK-3-mediated phosphorylation of β-catenin, the scaffold protein axin binds directly to both GSK-3 and β-catenin. Catenin is quickly degraded as a result of phosphorylation within this complex. As well as directly binding to axin and b-catenin, Adenomatous Polyposis Coli is necessary for the phosphorylation and destruction of β-catenin. According to the text, the pool of GSK-3 that is linked to the axin-APC complex is inaccessible to Akt and is consequently shielded from the insulin route. Axin-bound and insulin-regulated pools of GSK-3 are both directly inhibited by lithium, which causes lithium to activate intermediate effectors for both pathways Given the various ways that GSK-3 is regulated, it is conceivable to create inhibitors that block GSK-3 in a specific signalling pathway without impacting other signalling pathways.

As a result, a secondary mechanism that may increase the in vivo effectiveness of GSK-3 inhibitors enhances direct inhibition of GSK-3 (Beaulieu et al., 2008); (Huang et al., 2006). Direct GSK-3 inhibitors stop this process of auto-activation. (Fig. 21).

#### 7.6.2 CDK5 inhibitors

In some tau transgenic mice, pathogenesis seems to be influenced by CDK5 inhibitors, however the reported effects are small. CDK5 inhibitor use in humans has not yet been reported (Davies and Koppel, 2009).

#### 7.6.3 HSP modifiers

The Hsc70-interacting protein (CHIP)'s ubiquitin ligase c terminus can polyubiquitinate tau and may be a key factor in limiting the buildup of phospho-tau and NFTs. In several parts of the brain, including the cortex and hippocampus, CHIP-negative mice assemble phosphor-tau. However, NFT pathology is decreased in CHIP negative animals despite phosphotau accumulation, indicating that ubiquitination may be involved in tangle maturation.This suggests a possible method of reducing NFTs by avoiding polyubiquitination. A possible tactic is to trick cells into generating more heat shock proteins. Heat shock factor 1may be released from multichaperone complexes when Hsp90 inhibitors, for instance, cause the complexes to disintegrate. The fate of ubiquitinated tau can be modified by this transcription factor, which in turn can promote the de novo creation of heat shock proteins like Hsp70.(Dickey et al., 2008); (Schenk et al., 2006).

### 7.7 Antioxidants

It has long been understood that Alzhimers disease and the buildup of ROS in CNS cells are closely correlated. Numerous studies show that the direct effect of Aβ, activation of microglial cells, NFT, calcium, and metals all work together to produce ROS and RNS. The endogenous defence mechanism in nerve cells shields them from high ROS concentrations. This system ultimately decides how oxidative stress affects the cell and the body as a whole. The enzymes that detoxify ROS, like catalase and glutathione peroxidase (GTP), are the main components of the system. A nuclear transcription factor significantly controls how these enzymes function. NF-kB (Sorimachi et al., 1989; Lovstad, 1987).

Antioxidant molecules may be used as a prophylactic measure because of their capacity to neutralise unbound or improperly bound metals, preventing the 'down-stream' production of reactive oxygen species (Shishodia et al., 2005). In cell-based research, many substances with antioxidant capabilities, including oestrogen, melatonin, vitamin C and E, ginkgo bilboa extract, curcumin, and flavonoids, protect neurons from the damage caused by Aβ (Cheng et al., 2006, Zatta et al., 2003) and animal models (Feng et al., 2004); (Woo et al., 2009); (Oken et al., 1998); (Parks et al., 2001), but have shown conflicting results in a clinical setting (Zandi et al., 2004); Gómez-Ramos et al., 2004; (Lloret et al., 2009) and may even be detrimental (Judge et al., 2017); (Cruz and Tsai, 2004).

### 7.8 Therapies targeting neuroinflammation

Inflammation-related neurodegeneration has received a lot of attention during the past 15 years. When the Aβ and tau are present simultaneously, gliosis microgliosis and astrocytosis plays a significant part in the pathophysiology of AD. An innate immune expression that involve in complement activation (Bachiller et al., 2018), the formation of chemokines, the secretion of pro-inflammatory cytokines, and the excretion of nitric oxide which causes apoptosis, is linked to Aβ deposition and plaque development (Griffin et al., 1989); (Kontoghiorghes and Kontoghiorghe, 2020); (Davies and Koppel, 2009).

Epidemiological studies have also shown a significant decrease in the likelihood of AD development when NSAID use is long-term. Over the past ten years, a lot of clinical investigations have been conducted due to the extremely widespread usage of various NSAIDs and other anti-inflammatory medications. The Cache County Study on Memory Health and Ageing, a sizable population-based longitudinal study, recently raised the possibility that NSAID use, if begun in middle age, could help delay cognitive deterioration. Patients having one or more apolipoprotein E 34 alleles experienced the effect more visibly (Davies and Koppel, 2009; Vasak and Hasler, 2000).

The ability of PPARγ agonists to inhibit neuroinflammation makes them a significant additional class of drugs. The nuclear hormone receptor transcription factor known as PPARγ is ligand-dependent and controls inflammatory responses in a variety of organ systems, including the central nervous systemWhen engaged, PPARγ regulates the expression of specific genes for inflammatory mediators in T cells, including TNF, IL-10, IFN-, and IL-4, by binding to the peroxisome proliferator response element inside the promoter regions. In addition to inhibiting NF-kB-mediated inflammatory pathways by lowering its nuclear translocation, PPARγ activity also prevents Aβ from inducing microglial cells to produce pro-inflammatory cytokines (Ray and Lahiri, 2009).

### 7.9 Pharmacotherapies targeting metal ions

For many physiological processes, the equilibrium of metal ions (concentrations, distribution, stability, bioavailability) is essential. This is especially true in the CNS, where metals are crucial for the growth and maintenance of enzyme activity, mitochondrial function, neurotransmission, and memory. Because of the significance of metal ions, cells have created sophisticated machinery to control their equilibrium. The altered homeostasis of metal ions, however, can lead to a disease state, including a number of neurodegenerative illnesses, when these processes fail. Both under normal circumstances and during neurodegeneration, it is crucial to comprehend the structural and functional interactions of metal ions with the numerous intracellular and extracellular components of the CNS.As a result, the treatment approach of modulating metal ions has been suggested for AD and other neurodegenerative illnesses.Such treatment methods include antioxidants and metal-modulators (Gaggelli et al., 2006).

#### 7.9.1 Chelation therapy

After being incubated with chelators such trientine and D-penicillamine, the theory that metals are involved in the accumulation of A can be redissolved in aqueous form. In order to treat AD, Cherny et al. recommended that the usage of substances with metal chelation capabilities be taken into consideration. As a result, a current method for creating novel AD candidate treatments involves studying tiny compounds in vitro that can stop Aβ from aggregating, hence lowering its toxicity through metal complexation.

Metal chelators are substances that bind firmly to two or more metal ions to form a cyclic ring that converts the metal ions into inert forms. Desferrioxamine, a Fe chelator of this kind with a strong affinity for the metals Zn, Cu, and aluminium (Al), has been studied in clinical trials as a potential treatment for AD (Mandel et al., 2007). Chelators are substances that are made to attach to metal ions. By doing so, they become chemically inactive and incapable of contributing to the development of illnesses(Vasák & Hasler, 2000; (Nikseresht et al., 2019); (Hayakawa et al., 2003; Bjorkdahl et al., 2005).

#### 7.9.2 Metal complexes

Modulating metals with metal complexes is a further chelation technique. This strategy maintains the general homeostasis of metals by removing them from physiologically hazardous areas and maybe transferring them to areas where they are lacking. Metal-based compounds are showing promise as a novel AD treatment. Using pyrrolidine dithiocarbamate (M2 +-PDTC) or bis(thiosemicarbazone) (M2 +-BTSC) metal complexes, for example, to deliver Cu to cell regions deficient in the metal, or platinum (Pt) complexed to 1,10 phenanthroline derivatives (L-PtCl2) to prevent the detrimental binding of Cu to Aβ, are two examples of this strategy's rationale.Since PDTC, Cu, and/or Zn work together synergistically to inhibit transcription factor regulators of nuclear factor-κB (NF-κB), it is also known to have anti-inflammatory, antioxidant, and antiapoptotic properties (Furuta et al., 2002); (Flagstad, 1977).

Contrary to chelation, it is rational to take metals out of biologically hazardous regions and maybe transfer them to insufficient locations, maintaining the overall homeostasis of metals. Metal complexes can currently implement this strategy by either supplying metals to the adequare cell space using Metal-PDTC or Metal-BTSC, or by limiting metal-related harm to Aβ by combining platinum (Pt2 +) into 1,10 phenanthroline derivatives (L-PtCl2) (Vincent et al., 1997).

The anti-inflammatory and anti-apoptotic effects of this metal complex are brought about by the interaction of PDTC with copper and zinc. It prevents NF-kB from controlling transcription factors, activating Akt, and blocking GSK-3 in AD models (Cruz and Tsai, 2004; Gomez-Ramos et al., 2004).

#### 7.9.3 Metal-protein attenuating compounds

Due to MPACs' weak and reversible propensity towards metals, they can contend for metal ions with endogenous ligands. They are then able to identify and stop the harmful metal-protein reactions that are occurring "upstream." Because of this ability, they can revive normal metal levels in certain cellular compartments (Masters et al., 2006). Clioquinol served as the base for the first generation of MPACs. For many years, CQ was being used to treat dysentery and diarrhea along with anti-ambeic. CQ had been utilised as a therapy in cattle and humans with Zn-deficiency disorders (Kilic et al., 2012); (Fiehn, 2016).CQ is extremely lipophilic, rapidly absorbed, and capable of gluconation and sulfation. It can also penetrate the BBB and be excreted in the urine and faeces (Woo et al., 2011), (Liewendahl and Lamberg, 1967); (Zuccato and Cattaneo, 2009); (Gaggelli et al., 2006).

The specific mode of action for CQ is currently unknown. However, in cell culture studies it has been shown that CQ-copper complexes enter cells and significantly reduce the amount of Aβ that is secreted, which is thought to occur as a result of its breakdown through the upregulation of MMP-2 and MMP-3. Similar to metalbis (thiosemicarbazone), copper also encourages GSK-3 phosphorylation, which amplifies JNK activation and Aβ1–40 breakdown (Woo et al., 2011).

### 7.10 Apolipoprotein E (ApoE) inhibitors

The synthesis and secretion of apoE are significantly influenced by oestrogens, indomethacin, and probucol, according to in vitro *and* in vivoinvestigationson mature CNS rodents (mouse and rat). In addition to boosting apoE synthesis and release in vivo, oestrogen also promotes synaptogenesis and compensatory end remodelling in the mature hippocampus of mice in response to defecation. Indomethacin has been discovered to be a powerful inducer of apoE at subnanomolar doses. The component of the molecule responsible for the induction activity of apoE is different from the inhibitory moiety of cyclooxygenase, according to further investigation of the structural-activity relationship of a number of indomethacin derivatives. This suggests a non-immunosuppressive method of action that probably involves the peroxisome proliferating receptor-γ pathway in its active state. Similar to this, it has been discovered that after 30 days, the cortical and hippocampal regions significantly increase apoE synthesis and secretion. Probucol is an antiquated cholesterol-lowering medication for therapeutic management of familial hypercholesterolemia. In addition, probucol treatment was observed to increase synaptic density in 24-month-old rats as assessed by hippocampus GAP-43 and synaptophysin immunohistochemistry (Acharya et al., 2002; Catarina et al., 2010, Perry et al., 1987).

### 7.11 Other potential therapeutic strategies

#### 7.11.1 Drugs to target mitochondrial dysfunction

A novel method to treating AD that differs from the present body of knowledge, which is dominated by protein-focused approaches, targets organs (e.g. mitochondria).At the onset of AD, mitochondrial dysfunction can encourage synaptic loss and apoptosis and is thought to be a primary cause of neurodegeneration. It is possible for APP and Aβ to enter mitochondria where they converse with mitochondrial elements, limit ATP synthesis, and exacerbate oxidative damage (Greco et al., 2009).

#### 7.11.2 Neurotrophins

In reaction to injury, neurogenesis can take place in the adult brain. Recent studies have revealed a causal connection between an imbalance of NGF, activation of the amyloidogenic pathway.Neurotropin Growth Factor is necessary for the survival and fibre expansion of basal forebrain cholinergic neurons.NGF was specifically delivered to cholinergic basal brain neurons, where it reduced cell death, enhanced synaptic activity, and improved animal cognition. Initial research on AD patients used intracerebroventricular NGF infusion. Positive findings on cognitive and physiological tests of brain function were offset by negative side effects (such as pain and weight loss), which forced the administration of the intracerebroventricular medication to be stopped. Genetic therapy is a substitute approach that has been created:In a phase 1 research lasting 18 months, the delivery of NGF based on the intracerebral injection of genetically altered autologous fibroblasts to create human NGF was investigated in 8 patients with early AD.Despite the fact that two people experienced subcortical haemorrhages during implantation, it was shown that there was a decreased rate of cognitive decline and a higher rate of cortical glucose absorption (Greco et al., 2009; Goutte et al., 2002).

The primary regulators of synaptic plasticity, neuronal survival, and differentiation among these chemicals are brain-derived neurotrophic factors (Tapia-Arancibia et al., 2008) and safeguard against Aβ toxicity (Grundman et al., 2003). Neotrofin (AIT-082) is one instance, which has not demonstrated effectiveness in clinical trials (Douillet and Orgogozo, 2009).A selective 5-HT1A receptor antagonist and NGF inhibitor, xaliproden (SR57746)(Waddington, 2012). Whereas a subset of participants showed effects on hippocampal volume, two large 18-month trials failed to show clinical benefits in terms of cognition or overall dementia severity (Alvarez et al., 2006). In a number of randomised controlled trials, which lasted up to 28 weeks and involved individuals with mild to severe AD, significant impacts on cognition and overall evaluation were seen, but not on daily activities (Aisen et al., 2008); (Grimm et al., 2005).

#### 7.11.3 Statins

Statins can influence isoprenoids in addition to cholesterol via inhibiting HMG-CoA reduction. This latter point may be crucial to understanding how statins work because it has been demonstrated that they stimulate the expulsion of the ectodomain of sAPPα, which should limit the processing of APP by the β -and then γ- secretase. Statins also encourage the expulsion of the ectodomain of APP.

Even more complicated is how APP and lipids interact. The primary byproducts of γ-secretase, Aβ40 and Aβ42, can actually function as feedback to control lipid biology. According to available data, "Aβ40 inhibits HMG-CoA reductase and aids in maintaining low cholesterol levels, while Aβ42 activates sphingomyelinase and decreases sphingomyelin. Both cholesterol and sphingomyelin levels have been seen to rise in cells that have received a boost from γ-secretase (Giacobini, 1997). There is evidence from medicine that statins delay the development of AD, which is consistent with the clear complex interaction between lipids, statins, and APP processing (Schenk et al., 2006).

#### 7.11.4 The cholinergic approach to treatment of AD

Adults who develop dementia have memory and conceptualization problems are caused by of alterations in cholinergic function (DeTure and Dickson, 2019). The crucial role of cholinergic receptors in cognitive processes suggests that muscarinic central and nicotinic cholinergic receptors may have complicated interactions with learning and memory (X.-X. (Zhang et al., 2021; Waddington, 1942).

##### 7.11.4.1 Inhibition of ACh degradation

The most thoroughly investigated cognitive therapy for AD is comprised of AChE and ChE inhibitors. These medications have been shown to stabilise or temporarily slow the loss of cognitive ability as well as the symptoms of AD. They also temporarily improve or delay the symptoms of AD. When AChE, butyrylcholinesterases, and other cholinesterases are inhibited, ChE inhibitors are classified as non-specific inhibitors, or simply ChE inhibitors, or as specific inhibitors when AChE is the only cholinesterase they inhibit (Logsdon et al., 2007). Enzymes can either be irreversible, pseudo-reversible, or reversible depending on the degree of inhibition (Sano, 2000).

##### 7.11.4.2 Acetylcholine releasing agents

Different mechanisms can be used by a number of factors to affect ACh release. These include blocking potassium channels that are used to control the release of neurotransmitters, antagonistic interactions between receptors that control ACh release, and direct activation of Ca2 + channels to raise cytosolic Ca2 + and thereby cause the release of ACh (Goutte et al., 2002). Numerous additional substances, such as nootropics, can promote the release of neurotransmitters like ACh (Grundke-Iqbal et al., 1986a).

##### 7.11.4.3 Cholinergic drugs

Researchers have also looked into how nicotin receptor agonists can enhance cholinergic transmission. Ispronicline, a selective agonist of the α4β2 neuronal receptor, improves cognition in both healthy individuals and those with memory loss associated with age 46. In healthy volunteers, ABT-089, a partial agonist of the neuronal receptors α4β2 and α6β2, can reverse the cognitive deficit brought on by hyoscine however, in patients with mild-to-moderate AD enrolled in Phase 2 RCT were receiving treatment with a steady acetylcholinesterase inhibitor found no efficacy (Greco et al., 2009).

#### 7.11.5 Hormone replacement therapy

Estrogen lowers oxidative stress, increases blood flow to the brain, modifies the actions of nerve growth factors and inhibits cholinergic neuron atrophy (Janus et al., 2000). Additionally, it may reduce neuronal injury by preventing the production of Aβ. Postmenopausal oestrogen replacement treatment (ERT) has been demonstrated to be able to postpone the beginning of AD, according to three prospective population-based epidemiological investigations. A meta-analysis and randomised clinical trial both shown some improvements in cognitive function (Monsonego et al., 2003). However, other randomised studies have revealed no enhancement in cognitive or functional outcomes and an increase in dementia development in otherwise healthy individuals. Therefore, more investigation is undoubtedly required to determine whether ERT use in AD has direct or indirect effects. Using Estradiol (E2) as a therapy for AD, Fillit and colleagues published an open-label research in 1986 (Mehler, 2008).

In Randomised Controlled Trials (RCTs), antioxidants (like vitamin E) and omega-3 polyunsaturated fatty acids have been evaluated as alternatives to traditional treatment methods. Docosahexaenoic acid nutriment in older adults with cognitive impairment or AD has been shown to have positive effects in several studies (Greco et al., 2009; Zlokovic, 2005).

## 8 Summary

Summarising AD biomarkers and treatment available at present as:

**Table:** 

**Sr. No.**	**Drug Targets in Alzheimer’s Disease (AD)**	**Mechanisms, Current Treatment, Drug Therapy and Future Scope**
1	Amyloid β (Aβ)	Prevention of Aβ aggregation and clearance employing passive immunotherapy leading to inhibition. NABT-5102A, Scyllo-inositol are under trial
2	α-Secretase	Activation by agonists of muscarinic, glutamate, serotonin receptors, statins, estrogen, testosterones,proteinkinase C-activators. Etazolate is a clinically tested α-stimulator
3	β-Secretase	Inhibition of β-Secretase, ihibtors like Rosiglitazone, pioglitazone. CTS-2166 has entered trials.
4	γ-Secretase	Semagacestat is inhibitor of γ-Secretase in Phase-III clinical trials, MK0752 is in Phase-I clinical trials.
5	Tau Protein aggregation	Prevention of Tau aggregation
6	Tau Kinases	Inhibition of Tau Kinases, Kinases inhibitors like DYK1A, SRN-003–55, CHIR-98014,SB216763, Monoclonal Antibodies like Bapineuzumab, solanezumab, Gantenerumab and PF-0436036.
7	Glycogen Synthase kinase-3β	-Direct inhibitors: Prevents autoactivation -Indirect inhibitors: increases N-terminal phosphorylation
8	CDK-5 inhibitors	CDK-5 inhibition is reported in animal models but no reports are available in Humans
9	Heat Shock Proteins (HSP)	Increase in HSP production. Ubiquitin ligase C-terminus of HSC-70 interating protein (CHIP) prevents polyubiquitination. Fooling of Cells into producing more heat shock proteins.
10	Oxidative Stress Reactive Oxygen Species and Reactive Nitrogen Species (NOS)	Antioxidant therapy Antioxidants such as estrogen, melatonin, Vitamin C, E, Ginkgo biloba extracts, Curcumin, flavonoids, etc., proved to be useful in animal models but no confirmation is provided in humans.
11	Inflammation in AD	Treatment involving NSAIDS, PPARγ agonist Proinflammatory cytokinase
12	Metals in AD	Prevention, inhibition, and removal of metal ions -Chelation therapy: Trientine, D-penicillamine, Desferrioxamine(DFO) -Metal Complexes: PyrolidineDithiocarbamate, Phenanthroline derivatives -Metal Protein Attenuating Compounds(MPACs): Clioquinol derivatives
13	Apolipoprotein E (ApoE) Modulators	An increase in levels of ApoE prevents aggregation Estrogen, indomethacin, probucol enhances ApoE synthesis and secretion
14	Notch Signaling	BMS-708163 is used to treat Notch related adverse events.

## Compliance with ethical standards

This article does not contain any studies involving human participants performed by any of the authors.

## Funding

This research did not receive any specific grant from funding agencies in the public, commercial, or not-for-profit sectors.

## Conflict of interest

None.
